# Rational Development of a Carrier-Free Dry Powder Inhalation Formulation for Respiratory Viral Infections via Quality by Design: A Drug-Drug Cocrystal of Favipiravir and Theophylline

**DOI:** 10.3390/pharmaceutics14020300

**Published:** 2022-01-27

**Authors:** Si Nga Wong, Jingwen Weng, Ignatius Ip, Ruipeng Chen, Richard Lakerveld, Richard Telford, Nicholas Blagden, Ian J. Scowen, Shing Fung Chow

**Affiliations:** 1 Department of Pharmacology and Pharmacy, Li Ka Shing Faculty of Medicine, The University of Hong Kong, Pokfulam, Hong Kong, China; snwongab@connect.hku.hk (S.N.W.); irenewjw@connect.hku.hk (J.W.); igna1130@hku.hk (I.I.); 2 Department of Chemical and Biological Engineering, The Hong Kong University of Science and Technology, Clear Water Bay, Hong Kong, China; rchenar@connect.ust.hk (R.C.); kelakerveld@ust.hk (R.L.); 3 School of Chemistry and Biosciences, University of Bradford, Bradford BD7 1DP, UK; r.telford@bradford.ac.uk; 4 School of Pharmacy, University of Lincoln, Lincoln LN6 7TS, UK; NBlagden@lincoln.ac.uk; 5 School of Chemistry, University of Lincoln, Lincoln LN6 7TS, UK; IScowen@lincoln.ac.uk; 6 Advanced Biomedical Instrumentation Centre, Hong Kong Science Park, Shatin, Hong Kong, China

**Keywords:** cocrystal screening, inhalable cocrystal, drug-drug cocrystal, antiviral cocrystal, reformulation, quality-by-design, SARS-CoV-2, improved pharmaceutical properties

## Abstract

Formulating pharmaceutical cocrystals as inhalable dosage forms represents a unique niche in effective management of respiratory infections. Favipiravir, a broad-spectrum antiviral drug with potential pharmacological activity against SARS-CoV-2, exhibits a low aqueous solubility. An ultra-high oral dose is essential, causing low patient compliance. This study reports a Quality-by-Design (QbD)-guided development of a carrier-free inhalable dry powder formulation containing a 1:1 favipiravir–theophylline (FAV-THP) cocrystal via spray drying, which may provide an alternative treatment strategy for individuals with concomitant influenza infections and chronic obstructive pulmonary disease/asthma. The cocrystal formation was confirmed by single crystal X-ray diffraction, powder X-ray diffraction, and the construction of a temperature–composition phase diagram. A three-factor, two-level, full factorial design was employed to produce the optimized formulation and study the impact of critical processing parameters on the resulting median mass aerodynamic diameter (MMAD), fine particle fraction (FPF), and crystallinity of the spray-dried FAV-THP cocrystal. In general, a lower solute concentration and feed pump rate resulted in a smaller MMAD with a higher FPF. The optimized formulation (F1) demonstrated an MMAD of 2.93 μm and an FPF of 79.3%, suitable for deep lung delivery with no in vitro cytotoxicity observed in A549 cells.

## 1. Introduction

The pandemic caused by coronavirus 2 (SARS-CoV-2), which results in a severe acute respiratory syndrome, has raised an unprecedentedly high level of awareness towards influenza viral infections in history and resulted in over 2.6 million deaths according to the WHO as of March 2021 [1]. As a public health emergency of international concern, this incident has reignited attention about effective management of novel or re-emerging influenza infections. According to the Centers for Disease Control and Prevention (CDC) [2], individuals with certain underlying medical conditions, particularly chronic lung diseases (e.g., chronic obstructive pulmonary disease (COPD) and asthma), have been identified to show higher risk of being infected with SARS-CoV-2 and developing more severe pneumonia and acute respiratory failure, which are likely to be the leading causes of death. A number of clinical analyses supports this correlation [3,4,5,6,7,8,9,10,11]. For example, it has been reported that COPD patients have a 4-fold higher risk of SARS-CoV-2 infection than the healthy population [12]. The impaired lung function and marked airway inflammation induced by COPD/asthma are considered as poor prognostic factors when SARS-CoV-2 infection is presented [13]. Stabilizing such conditions is therefore a crucial treatment strategy to minimize the infection risk. These highlight an urgent need to establish an effective pharmacological intervention in preventing and treating viral respiratory infections for these vulnerable patient groups.

Since SARS-CoV-2 primarily attacks the lungs, pulmonary delivery of antiviral agents is highly desirable to achieve targeted therapy. In the context of localized treatment for respiratory diseases, inhalation of aerosols can maintain a high drug concentration in the inflamed lung endothelial cells and result in rapid onset of action for viral eradication and enhanced therapeutic efficacy, making dose reduction possible. In addition, the respiratory tract provides a less harsh and low enzymatic environment with minimal hepatic first-pass metabolism [14]. These unique hallmarks render pulmonary delivery an attractive non-invasive alternative route of administration for delivering small molecules and biological therapeutics for various potential treatments, such as antiviral, antibacterial, anti-inflammatory, anti-asthma, anti-hypertensive, and anticancer [14,15,16,17,18,19,20,21,22,23,24,25]. However, it should be noted that most of the marketed antiviral drugs are administered orally, which implies a significant proportion of drug is distributed to systemic circulation besides the lung.

Favipiravir (FAV) is a substituted pyrazine derivative indicated for the treatment of novel or re-emerging pandemic influenza virus infections when the patient is refractory or not responsive to other anti-influenza viral agents [26] (Figure S1). It recently showed promise in preclinical and clinical trials for the pharmacological activity against SARS-CoV-2 [27,28,29,30,31,32,33,34], whereas only a very high oral dose could significantly reduce viral replication at the infection site, mainly due to its low aqueous solubility at pH 2.0 to 6.1, i.e., 2.29 mg/mL at 20 °C (Table S3) [35,36,37]. This raises toxicity concerns [27,38] and low patient compliance associated with a high pill burden for the vulnerable population, especially frail elderly and those with multicomorbidity. To this end, the delivery of an inhaled FAV formulation for direct lung targeting appears as a rational approach to surmount the disadvantages as mentioned earlier.

Different inhalation devices are specifically designed for generating drug aerosols. Among them, dry powder inhalers (DPIs) are propellant-free and portable devices utilizing the patient’s inspiratory flow for aerosol dispersion and entrainment into the lungs [39]. It is widely recognized that particles with an aerodynamic diameter between 1 and 5 μm are ideal for deep lung delivery [40]. However, these micronized drug particles confer a high level of surface free energy, resulting in a strong propensity to be retained in the inhaler [41]. In order to circumvent the poor flowability and dispersibility of particles, drug carriers such as lactose and mannitol are commonly added into the formulation with a typical drug-to-carrier ratio of 1:67.5 *w*/*w* [41,42,43]. Nonetheless, the incorporation of excipients often leads to issues related to blend uniformity and limited drug loading. A significant challenge is encountered in delivering a high dose of drugs to the patients [44]. The development of high-potency DPI formulation with absence of carrier is thus desired, and has become a subject of active research in recent decades [41,45,46,47,48].

The advent of pharmaceutical cocrystals, an emerging class of crystalline material making the revival of old or problematic drugs viable, offers a previous opportunity to achieve such purpose. The assumption lies in the fact that a cocrystal can modulate a series of pharmaceutical properties, such as dissolution, stability, hygroscopicity, mechanical properties, and bioavailability, etc. [49,50,51,52,53,54,55], and thereby potentially replacing the function of conventional excipients. It is worth mentioning that the majority of cocrystal research has been focusing on the oral dosage forms over the past two decades. In contrast, effort devoted to the development of inhalable drug-drug cocrystal for combination therapy is scarce [54]. This unique niche deserves more attention since significant clinical demand in handling the growing global prevalence of respiratory diseases is foreseeable due to population aging and increased exposure to high air pollutants.

In the present work, we aimed to develop a carrier-free favipiravir–theophylline cocrystal DPI formulation, which potentially constitutes an effective prophylaxis and treatment strategy against novel and/or re-emerging mild-to-moderate influenza viral infections in high-risk patients with existing COPD or asthma. Theophylline (THP), a non-selective phosphodiesterase (PDE) inhibitor given systemically as oral tablets [56], was chosen owing to its bronchodilator and anti-inflammatory properties (Figure S1). THP has a long medical history for the treatment of COPD and asthma worldwide with a relatively low cost. However, the treatment regimen is complicated by its poor aqueous solubility, i.e., 7.14 mg/mL at 20 °C (Table S3), and extensive first-pass metabolism [57,58]. Use of systemically delivered PDE inhibitors has been restricted by systemic adverse effects. Oral THP appears to be facing obsolescence when inhaled b2-agonists and inhaled corticosteroids with better tolerance and efficacy are being introduced to the market [59,60]. This provides a good clinical rationale to formulate PDE inhibitors as inhaled form, although advancing such formulation through clinical development seemed an obstacle [61]. Furthermore, in supramolecular chemistry, THP has been reported to cocrystallize with several coformers containing primary amides (e.g., urea, formamide, and pyrazinamide) via formation of an amide-pseudo-amide synthon between the amide coformer and the HN−C−C=O moiety of the THP molecule [62]. Hence, it could be postulated that FAV, which contains a pyrazine structure, would have a high propensity to form a cocrystal with THP.

We herein employed spray drying to prepare an inhalable FAV-THP cocrystal dry powder. Followed by the characterization of the physicochemical properties of the cocrystal in comparison to its parent drugs, the in vitro aerosolization behavior under different inhalation flow rates and cytotoxicity were also examined. We anticipate that co-spray drying multiple active pharmaceutical ingredients (APIs) as a cocrystal entity is a promising strategy for developing an excipient-free DPI formulation which offers simultaneous improvement in aerosol performance and physicochemical properties of problematic drugs. Previous work by Alhalaweh et al. reported the production of theophylline cocrystals with urea, saccharin, and nicotinamide as the coformers via spray drying, suggesting the possibility of obtaining highly crystalline inhalable cocrystal with different particle characteristics [63]. However, the precise correlation between drug physicochemical properties, spray drying process parameters, and the aerosol performance of the cocrystal-based DPI system remains obscure. Such information is essential from the viewpoints of production, quality control, and regulatory. Hence, we adopt a Quality by Design (QbD) approach to elucidate how different critical process parameters (CPPs) of spray drying would dictate the integrity and aerosolization performance of the resultant inhalable cocrystal formulations. To our knowledge, it is the first time that QbD is utilized to guide the design, manufacture, and optimization of an excipient-free drug-drug cocrystal DPI formulation. In long term, this study also exemplifies the clinical value of applying spray drying to prepare cocrystals comprising different APIs. Such integrated inhaled delivery platform comprising modern crystal engineering and sophisticated particle engineering could eventually benefit the development of personalized medicine and be translated to different patient groups suffering from multiple pulmonary comorbidities.

## 2. Materials and Methods

### 2.1. Materials

FAV (≥98%) was purchased from Yick Vic Chemicals & Pharmaceuticals Limited (Hong Kong, China). THP (≥99%), sodium chloride (NaCl, ≥99%), and potassium chloride (KCl, ≥99%) was supplied by Sigma-Aldrich (St. Louis, MO, USA). Potassium dihydrogen orthophosphate (KH_2_PO_4_, ≥99.5%), sodium dihydrogen phosphate monohydrate (Na_2_HPO_4_, ≥98%), and ethanol of analytical grade were sourced from VWR BDH Chemicals (VWR International S.A.S., Fontenay-sous-Bois, France). Acetonitrile (ACN) and isopropanol (IPA) of analytical grade were obtained from Merck KGaA (Darmstadt, Germany). Potassium bromide (KBr) for FTIR analysis was obtained from J&K Scientific Limited, China. Water was purified through a Thermolyne NANOpure Diamond Analytical ultra-pure water system (Barnstead, Thermo Fisher Scientific, Waltham, MA, USA). Dulbecco’s Modified Eagle Medium: Nutrient Mixture F-12 (DMEM/F-12), fetal bovine serum (FBS), antibiotic-antimycotic (100×), 0.25% (*w*/*v*) trypsin-EDTA solution, and 3-(4,5-dimethylthiazolyl-2)-2,5-diphenyltetrazolium bromide (MTT) were purchased from Thermo-Fisher Scientific (Waltham, MA, USA).

### 2.2. Implementation of Quality by Design (QbD)

A three-factor, two-level, full factorial design with proper randomization was used to optimize the manufacturing process of FAV-THP cocrystal DPI formulations. Critical process parameters (CPPs) and critical quality attributes (CQAs) were initially identified. Three CPPs, namely X_1_: total solute concentration (concentration of dissolved FAV and THP, mg/mL), X_2_: feed pump rate (mL/min), and X_3_: atomizing air flow (L/h), ought to be considered in the development of inhalable dry powder for efficient pulmonary delivery. The inlet temperature was fixed at 80 °C. The levels of each variable were denoted as −1, 0 and +1, and their corresponding values are shown in Table 1. The range of the levels was set based on the reported works [63,64,65]. The following CQAs were selected as responses: Y_1_: mass median aerodynamic diameter (MMAD, μm), Y_2_: fine particle fraction (FPF, %), Y_3_: emitted fraction (EF, %), Y_4_: crystallinity index (CI, %). The factorial design allows to investigate the influence of selected CPPs on the CQAs of the spray dried cocrystal powder. A total of 11 formulations were produced as described in Table 2. The center point (CP) was run in triplicate to evaluate the curvature and precision of the production process.

### 2.3. Preparation of FAV-THP Cocrystal

Attempts to cocrystallize FAV with THP were made using rotary evaporation and spray drying. Equimolar amounts (0.89 mmol) of FAV (139.70 mg) and THP (160.30 mg) were dissolved in ethanol, followed by sonication until a clear solution was obtained. Preliminary cocrystal screening was carried out using a rotary evaporator (Buchi, Germany) under a vacuum with the rotary flask being immersed in a water bath at 60 °C. The resulting product was oven-dried at 60 °C for 3 h and gently triturated to a fine powder for further analysis. For the production of inhalable FAV-TPH cocrystal formulation, the solution was spray-dried using a Büchi B-290 spray dryer with a B-296 Dehumidifier and B-295 Inert Loop (Büchi Labortechnik, Flawil, Switzerland). Nitrogen was used as the drying gas. A total of 11 formulations of FAV-THP cocrystal powder were prepared under the conditions as listed in Table 2. The CPPs, (i.e., solute concentrations, feed rates, and compressed gas atomization flow rates) were examined, while other processing parameters were fixed: inlet temperature of 80 °C, and aspiration at approximately 35 m^3^/min [63,66]. The resulting outlet temperature varied from 48–58 °C (Table S1). The final products were stored in tightly sealed collectors until further analysis.

### 2.4. Single Crystal X-ray Diffraction

**Figure 1:**
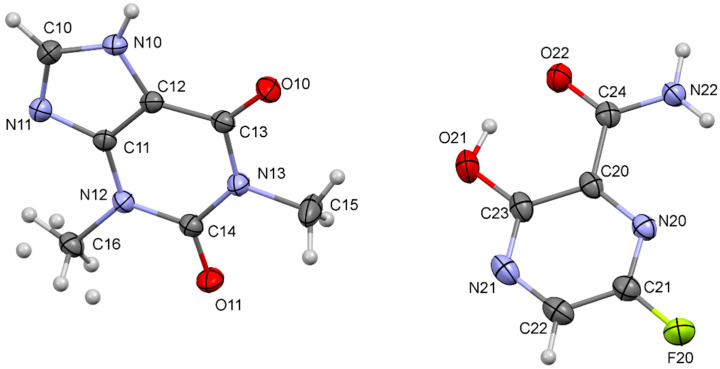
Thermal ellipsoid plot of the asymmetric unit of THP:FAV 1:1 showing the numbering scheme for the single crystal structure.

Colorless single crystals were tested from three separate batches (batches 1–3) and each were shown to be isomorphous, corresponding to the cocrystal form. A full set of single crystal diffraction data were obtained for a colorless lath (0.296 × 0.220 × 0.076 mm^3^) selected from batch 2 with a Bruker Advance diffractometer and Photon III-14 CMOS detector at 173 K using Bruker APEX3 software. The structure was solved by direct methods (SHELXS) revealing the position of all non-hydrogen atoms in the structure and all hydrogen atoms were located with subsequent difference-Fourier synthesis of the data. Structure was refined by full-matrix least squares on *F*^2^ with anisotropic displacement parameters assigned to all non-hydrogen atoms. The structure of the asymmetric unit is given in Figure 1. With the exception of the methyl hydrogens on C16, positional parameters of all hydrogen atoms in the structure were freely refined along with their respective isotropic displacement parameter. Hydrogens at C16 were disordered over two sites with occupancy fixed at 0.65:0.35—positions and displacement parameters refined acceptably for the major component—while the located positions of minor component were fixed in subsequent refinement cycles. A small correction for extinction was applied along with a weighting scheme to the data: *w* = 1/[σ(*F*_o_^2^) + (0.0550*P*)^2^ + 0.56*P*] where *p* = [max (*F*_o_^2^, 0) + 2*F*_c_^2^]/3. Refinement converged at *R*_1_ = 0.0356 [*F*_o_ > 4σ(*F*_o_)] and *wR*_2_ = 0.0974 (all data). Structural details are deposited with the Cambridge Crystallographic Data Centre (CCDC) with reference number 2087671.

### 2.5. Powder X-ray Diffraction (PXRD)

The X-ray powder diffraction data was collected using a Panalytical X-ray diffractometer (Philips X’Pert PRO, Eindhoven, The Netherlands), equipped with Cu−Kα radiation (λ = 1.5406 Å, 40 kV, 40 mA). A sample was evenly packed in a custom-made aluminum holder with a 2 mm depth and scanned from 2θ interval of 5–35° at 0.04° step size with 4° per minute scanning speed. The crystallinity index of different spray-dried cocrystal formulations prepared by the DoE method was calculated using the OriginPro (OriginLab Corporation, Northampton, MA, USA) as previously reported [67].

### 2.6. Thermal Analysis

Differential scanning calorimetry (DSC) and thermogravimetric analysis (TGA) profiles were generated by a TA DSC 250 differential scanning calorimeter (TA Instruments, New Castle, DE, USA) and a TGA Q5000 thermogravimetric analyzer (TA Company, New Castle, DE, USA), respectively. For DSC experiments, pure indium was used for routine calibration of enthalpy and cell constant. A weighed sample (~3 mg) was encased in a Tzero Aluminum Hermetic pan (TA Instruments, New Castle, DE, USA) with pinhole-vented lid if required and heated from 50 °C to 300 °C at a scanning rate of 10 °C/min to generate the thermogram. In the TGA experiments, each sample (5–7 mg) was placed on an open pan and heated at 10 °C/min from 50 °C to 300 °C. Nitrogen was used as the purge gas at 20 mL/min for both the DSC and TGA analyses. The TA Trios Software was used for data analysis.

### 2.7. Dynamic Vapor Sorption (DVS)

Water sorption isotherms of the samples were obtained using an automated vapor sorption analyzer (DVS Advantage-1, Surface Measurement Systems Ltd., Allentown, PA, USA) at 25 °C (±0.1 °C) and a total nitrogen flow of 200 cm^3^/min. The sample (ca. 50 mg) was first purged with dry nitrogen to constant weight. Subsequently, the sample was exposed to relative humidity (RH) in the range 0% to 95% with a step size of 5% RH, returning to 0% RH with the same 5% step size on completion of the initial cycle. The RH was changed to the next target value once the equilibrium was reached, where either dm/dt was ≤0.002% with a minimum equilibration time of 0.5 h or maximum equilibration time of 6 h at each specific RH.

### 2.8. Fourier-Transform Infrared Spectroscopy (FTIR)

The FTIR spectra were obtained with an FTIR spectrophotometer (Spectrum Two, PerkinElmer Instrument, Norwalk, CT, USA) in a KBr diffuse reflectance mode. The scan was performed in the range of 4000 cm^−1^ to 400 cm^−1^ at an interval 0.5 cm^−1^. A total of 32 scans were collected at a resolution of 4 cm^−1^ for each sample.

### 2.9. Particle Size Distribution Measurement by Laser Diffraction

The volumetric size distribution of the formulations was determined by the HELOS/KR laser diffractometer with an INHALER module (Sympatec, Germany) for sizing the particles after dispersing from a Breezhaler^®^. Approximately 5 mg of powders were dispersed through the laser measurement zone at 1 bar of air pressure. The airflow rate was set to 60 L/min, which generates a 1.5 kPa pressure drop across the inhaler. The measurement was conducted with a 100 mm (R3) lens (measuring range 0.45–175 μm). The volume particle size data (D10, D50 and D90) corresponding to the equivalent spherical volume diameters at 10%, 50% and 90% cumulative volume was obtained. The width of the distribution, i.e., SPAN, was calculated as (D90 − D10)/D50.

### 2.10. Scanning Electron Microscopy (SEM)

The particle morphology of the samples was observed by field emission scanning electron microscopy (Hitachi S-4800 FEG, Hitachi, Tokyo, Japan). The powders were sprinkled onto carbon adhesive tape mounted on SEM stubs. Any sample not adhering to the tape was removed by compressed air. A sputter coater (Bal-tec SCD 005 Sputter Coater, Bal-Tec GmbH, Schalksmühle, Germany) was used to coat the powder with approximately 11 nm gold-palladium alloy in two cycles (60 s each) to create a conductive layer and avoid overheating.

### 2.11. High Performance Liquid Chromatography (HPLC)

The concentrations of FAV and THP were quantified by HPLC equipped with a diode array detector (Agilent 1200 series, Agilent Technologies, Wilmington, DE, USA) and an Agilent Zorbax Eclipse Plus C18 column (5 μm, 250 mm × 4.6 mm) in an isocratic condition. The mobile phase consisted of a mixture of 10% acetonitrile and 90% 50 mM KH_2_PO_4_ buffer solution adjusted to pH 7 with NaOH. The detection wavelengths were 360 nm and 280 nm with retention times at 3 min and 7 min for FAV and THP, respectively. This method was sensitive to a lower limit of quantitation of 0.1 μg/mL, and validated for linearity (R^2^ = 0.9999 for FAV and 0.9997 for THP) over the linear range 0.1–150 μg/mL. A 25 μL aliquot of each sample solution was injected and ran at a flow rate of 1 mL/min.

### 2.12. In Vitro Aerosol Performance Evaluation

The in vitro aerosol performance of the spray dried FAV-THP cocrystal powder formulations was determined with a Next Generation Impactor (NGI, Copley, Nottingham, UK). A thin layer of silicon grease (Slipicone; DC Products, Waverley, VIC, Australia) was coated onto the impactor stages prior to dispersion to prevent particle bounce. Approximately 5 mg of powders were loaded into a size 3 hydroxypropyl methylcellulose capsules (Capsugel, West Ryde, NSW, Australia), which were aerosolized by Breezhaler^®^ (Novartis Pharmaceuticals, Hong Kong, China). The powders were dispersed at a flow rate of 60 L/min for 4 s. Since the patients with co-existing COPD and influenza viral infection may have reduced lung function, the aerosol performance of the optimized FAV-THP cocrystal powder was further tested under lower inhalation flow rates (30, 40, and 50 L/min) (Table S2). Different volumes of ethanol were used for rinsing and dissolving FAV and THP from all stages to allow the measurement of a quantifiable concentration: 5 mL for capsule, inhaler, adaptor, induction port (throat) and NGI Stage 1 to 4; 3 mL for NGI Stage 6 to 8. The solutions were subsequently filtered by 0.45 μm nylon syringe filters and assayed by HPLC. The recovered dose, emitted fraction (EF), fine particle fraction (FPF), mass median aerodynamic diameter (MMAD), and geometric standard deviation (GSD) were calculated using a previously published method [64]. The EF referred to the fraction of powder that exited the inhaler to the total recovered dose. The FPF was the mass fraction of the particles <5 μm with respect to the recovered dose. The recovered dose was defined as the sum of powder mass assayed on all the parts in a single run.

### 2.13. Dissolution Study

The dissolution performance of the optimized spray-dried FAV-THP DPI formulation (F1) was assessed in triplicates using a jacketed beaker, which contained 100 mL of simulated lung fluid (SLF3) as dissolution medium according to a reported protocol [68]. The pH 7.4 SLF3 was composed of 0.2033 g/L magnesium chloride, 6.0193 g/L sodium chloride, 0.2982 g/L potassium chloride, 0.071 g/L sodium sulfate, 0.3676 g/L calcium chloride dihydrate, 0.9526 g/L sodium acetate, 2.6043 g/L sodium hydrogen carbonate, 0.097 g/L sodium citrate dihydrate, and 0.142 g/L sodium phosphate monobasic monohydrate [69]. A Fast Screening Impactor (FSI; MSP Corporation, Shoreview, MN, USA) was used to collect the respirable fraction of the DPI formulation with an MMAD < 5 μm as described before [64,65]. In brief, a 10 mg of powders was loaded in a size 3 HPMC capsule, followed by dispersion at 60 L/min flow rate for 4 s, which generates a 1.4 kPa pressure drop across the Breezhaler. After dispersion, the powders with aerodynamic diameters <5 μm were deposited onto a glass fiber filter (ADVANTEC; Toyo Roshi Kaisha, Ltd., Tokyo, Japan) were transferred into the jacketed beaker. The dissolution test was carried out under sink condition at 37 °C and the medium was stirred at 75 rpm with a magnetic bar. The dissolution profile of equivalent mass of unformulated FAV and THP powders blended as physical mixture was also tested for comparison. A 1 mL of the dissolution medium was withdrawn at designated time points, i.e., 2.5, 5, 7.5, 10, 15, 20, 30, 45, and 60 min, and replaced with an equal volume of fresh medium. The sample solution was filtered through 0.45 μm nylon syringe filters and assayed by the HPLC.

### 2.14. Solubility Study

The aqueous solubility was determined by adding excess solid in screw capped test tubes with 3 mL of deionized water and shaking for 72 h at 20 °C. Samples were filtered through 0.45 μm membrane filters, followed by dilution to appropriate concentrations for the HPLC assay.

### 2.15. Stability Study

Raw FAV, raw THP, and the optimized FAV-THP spray dried cocrystal powder were stored in screw-capped glass bottles separately under 60 °C at 30% RH for 1 month. To assess their physicochemical stability under thermal stress, the samples before and after the storage were collected for PXRD and DSC analysis. The assay of drugs was quantified by HPLC.

### 2.16. MTT Cell Viability Assay

The A549 cells (human alveolar epithelial adenocarcinoma) were obtained from ATCC (Manassas, VA, USA) and cultured in DMEM/F-12 supplemented with 10% (*v*/*v*) FBS and 1% (*v*/*v*) antibiotic–antimycotic in a humidified incubator at 37 °C with 5% CO_2_. On the day before the addition of treatments, A549 cells were seeded in 96-well plates at a density of 2 × 10^4^ cells/well. Raw FAV and THP, FAV and THP physical mixture, and the spray-dried FAV-THP formulation F1 were dissolved in DMSO and subsequently diluted with complete DMEM/F-12 to concentrations of 1.6–1000 µM. After 24-h incubation with the treatments, the cells were incubated for another 3 h in the MTT solution (0.8 mg/mL). Then, the insoluble formazan was dissolved in IPA, and the absorbance at 570 nm was measured. Cell viability (%) was expressed as the percentage of the absorbance from the cells in the treatments against the absorbance from the cells in the complete DMEM/F-12 with the same concentration of DMSO as the treatment. All groups were repeated three times and each time in triplicates.

### 2.17. Statistical Analysis

The statistical analysis for DoE was performed using the Minitab^®^ 20 (Minitab Inc., State College, PA, USA) by applying one-way analysis of variance (ANOVA). The significant factors affecting each CQA were analyzed in Pareto charts with the aid of normal probability plots. Any terms crossing the reference line in a Pareto chart are statistically significant. A series of 2D Contour plots and 3D surface plots were constructed to determine the proven acceptable range of CPPs and establish the design space. A *p*-value < 0.05 was considered as statistically significant.

## 3. Results and Discussion

### 3.1. Cocrystallization of Favipiravir with Theophylline

**Figure 2:**
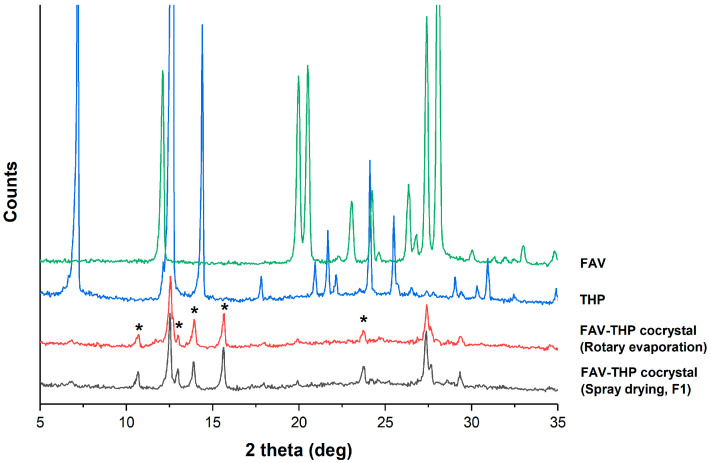
PXRD patterns of the FAV-THP cocrystal system. New characterization peaks of the cocrystal are marked with *.

A drug-drug cocrystal of FAV-THP was successfully obtained in a 1:1 stoichiometric ratio from ethanol through both rotary evaporation and spray drying. The PXRD patterns of the prepared samples exhibited a number of unique diffraction peaks (2θ = 10.71°, 12.98°, 13.91°, 15.66°, and 23.71°), while the characteristic peaks corresponding to FAV (2θ = 12.09°, 19.98°, and 20.51°) and THP (2θ = 7.18°, and 14.40°) were absent (Figure 2).

**Figure 3:**
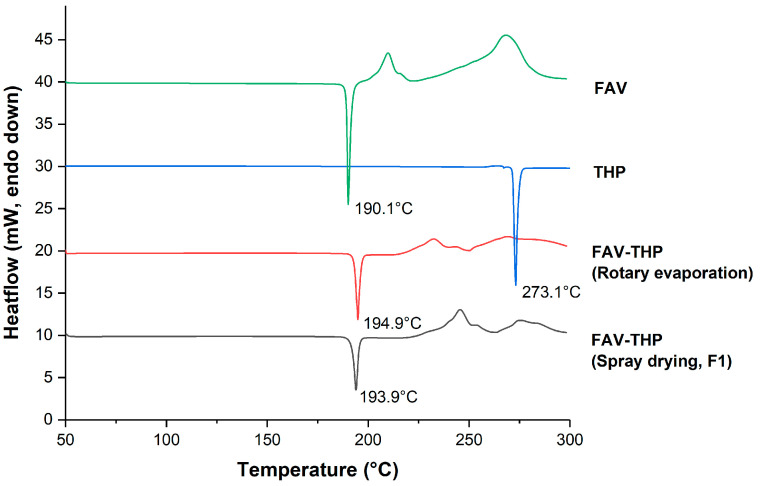
Overlaid DSC thermograms of the FAV-THP cocrystal system.

The overlaid DSC thermograms (Figure 3) indicated that the 1:1 FAV-THP cocrystals exhibited a sharp melting endotherm at 194.9 °C in between those of FAV (190.1 °C) and THP (273.1 °C), followed by thermal decomposition. This excludes the possibility of forming eutectic mixtures. The crystal lattice strengthening effect upon cocrystallization was observed by the elevated fusion enthalpy (ΔH_f_) of the FAV-THP cocrystal (37.50 kJ/mol) compared with its constituted components (FAV: 31.03 kJ/mol, THP: 31.62 kJ/mol). A relative humidity stress study and a thermal stress study were conducted to evaluate the stability of the formulation. The cocrystals remained stable at 60 °C for 1 month, without phase transformation detected during storage based on the DSC thermogram (Figure S2). As indicated by the DVS results, the amounts of moisture sorption of all cocrystal samples were not significant under 80% RH at 25 °C as the mass only changed less than 3%, therefore being non-hygroscopic. As expected, the FAV-THP cocrystals produced by spray drying exhibited higher moisture sorption than that produced by solvent evaporation (Figure S3). This could be attributed to a finer particle size and higher porosity of the spray dried powders, leading to a larger specific surface area where vapor sorption can occur [70]. For further examination of the new phase, a temperature-composition phase diagram was constructed using binary mixtures of cocrystal formers through DSC analysis (Figure S4). The phase diagram for the FAV-THP cocrystal system showed a local maximum melting temperature at 0.5 mole fraction of either cocrystal former, which confirmed its 1:1 stoichiometry. Two eutectic points were located at 0.38 and 0.8 FAV mole fractions with eutectic melting at 176.3 and 192.8 °C, respectively. In the TGA curve of cocrystal produced by both rotary evaporation and spray drying (Figure S5), the weight loss occurred at a temperature range of 145.6 to 234.1 °C, estimated as 46.5%. This is reasonably in line with the calculated value of 47.1% expected for a 1:1 stoichiometry, suggesting the accompanying loss of one molar equivalent of FAV from the 1:1 cocrystal lattice.

**Figure 4:**
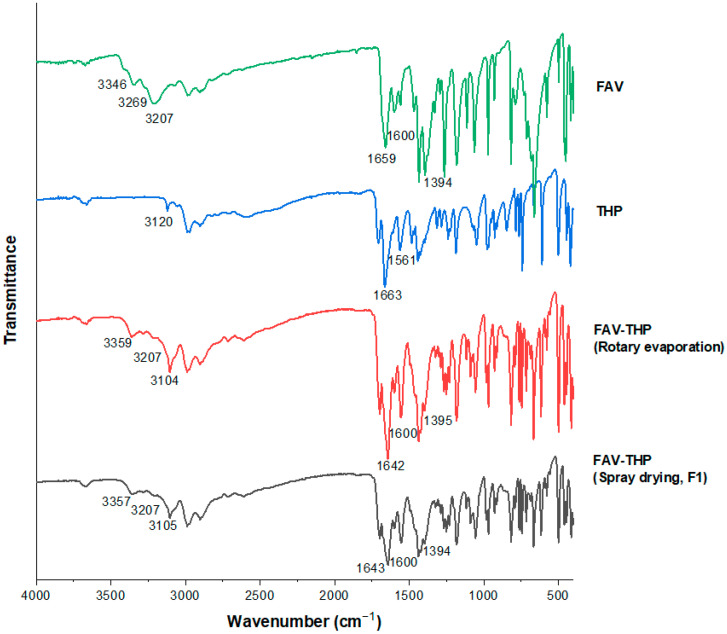
FTIR spectra of the FAV-THP cocrystal systems.

The FTIR spectra for FAV-THP cocrystal system produced by both rotary evaporation and spray drying are illustrated in Figure 4. The FAV-THP cocrystal obtained from the two methods shared essentially the same IR characteristic peaks. Spectral peak shifts were observed for various polar functional groups compared with parent drugs, as summarized in Table 3. A complex band region at 3450–3030 cm^−1^ in FAV-THP corresponds to the superposition of *ν*(NH_2_) and *ν*(OH) vibrations. The peak attributed to O−H stretching dramatically shifted from 3207 cm^−1^ to 3104 cm^−1^. The lower frequency implies the involvement of the O−H group in an intermolecular hydrogen bond without proton transfer, indicating the formation of a new phase.

### 3.2. Single Crystal X-ray Diffraction

Single crystal structural analysis confirmed the formation of a 1:1 cocrystal of THP-FAV. THP displays a challenging polymorphic landscape with five different forms [71,72]; with refcodes BLPLOT01 to BABPLOT06, DUWXEA, and KOJNIJ, and a hydrate forms, with refcodes THEOPH02 to THEOPH03. For simplicity, refcodes are used in this work. For FAV, refcode is DOHVED. As of 2021, the CSD database reports 88 adduct crystal forms, and these primarily utilize the dimer or chain assembly reported for the polymorphic landscape. This indicates the robustness of THP as a coformer. However, a similar search for FAV as of 2021, the CSD reports no adduct forms as this molecule as a coformer. However, very recent report of a similar THP-FAV cocrystal has emerged [73] but we can offer a more detailed view of crystal packing consistent with the robustness of this phase under the range of formulation conditions described.

**Figure 5:**
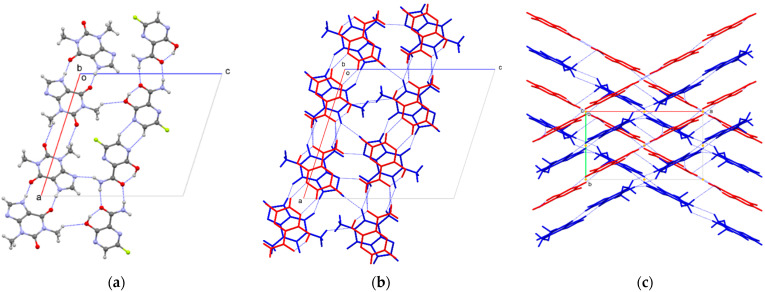
The crystal packing of THP:FAV showing (**a**) centrosymmetric hydrogen bonding ring motifs generating chains of THP and FAV viewed down the *b*-axis; (**b**) ring-stacking interleaving THP (blue) and FAV (red) chains (viewed down the *b*-axis) cross-linked by short contacts between THP:FAV imidazole-amide (>N…HN) and methyl-phenol (CH…OH), and by THP-THP carbonyl-imidazole (CO…HC) which propagate the structure parallel to *c*-axis; (**c**) the stacked interleaved chains of THP (blue) and FAV (red) chains viewed down the *c*-axis ‘crossing’ through the action of the 2_1_-screw axis parallel to *b*.

The principal structural components of this THP-FAV cocrystal can be viewed as discrete chains of THP and FAV, each formed from homo-molecular centrosymmetric hydrogen bonding motifs (Figure 5a) that interleave and cross-link to develop the overall crystal packing (Figure 5b,c). The FAV chains are formed through two separate centrosymmetric H-bonded rings: an R2,2(8) arising from amide…amide interaction and an R2,2(6) motif from CH···N between adjacent pyrazine rings. The THP chains are formed from two further centrosymmetric rings: R2,2,(10) motif between imidazole NH and carbonyl, and R2,2,(10) between N-methyl CH and carbonyl. The respective chains alternate in stacks parallel to the *b*-axis of the unit cell through FAV···THP π-π interaction over ca. 3.1 Å to form interleaved layers throughout the structure (Figure 5b). These layers are ‘crosslinked’ by short contacts: THP-FAV imidazole-amide (>N···HN) and methyl-phenol (CH···OH), and by THP-THP carbonyl-imidazole (CO···HC). These alongside Van der Waals interactions, propagate the structure along to *c*-axis (Figure 5b,c). The THP-THP chain closely resembles that formed in the reported anhydrous polymorph of THP (CSD Code: BAPLOT03), postulated to be the most stable anhydrate form of THP [74]. The structural analogy with this cocrystal is marked. FAV chains are inserted into the THP layers. This overall arrangement may correspond with the robustness of the formation of this THP-FAV cocrystal under the range of formulation conditions studied herein.

The crystal packing similarity wizard (standard settings with molecular cluster 6, distance and angle tolerance 5%, ignoring the smallest molecule component) was further employed to identify similarity in molecular packing environments within crystal structures. The 1:1 FAV-THP cocrystal contains a repeating pair of THP then FAV dimers, denoted AA:BB. It showed packing similarity with BABPLOT 02, BABPLOT 03, and KIGLUI 01 of 0.129%, 0.118%, and 0.139%, respectively. All these structures display dimeric AA-THP assemblies, and an AA:BB type assembly was observed in KIGLUI 01, which is characterized by a hydrogen bonding between the respective AA and BB pairings [75].

### 3.3. Application of DoE to the Process Optimization of Spray-Dried Pharmaceutical Cocrystal Dry Powder Formulation

#### 3.3.1. In-Vitro Aerosol Performance of Inhalable FAV-THP Cocrystal Powders

One specific advantage of spray drying lies in its ability to control both the solid-state and particle properties of inhalable products in one step, such as particle size, particle morphology, flowability, and dispersibility, etc. [63,76,77]. Process optimization facilitates the design of a more efficient drying condition and provides better understanding to secure the target product quality attributes to be fully fulfilled. While a number of studies have been dedicated to exploring the effects of spray drying processing parameters on product performance, none has correlated them to inhalable cocrystal production. In the present study, QbD was applied for this purpose. The aerosol performance of the spray-dried FAV-THP cocrystal powders was characterized by the MMAD, GSD, FPF, and EF. Their crystallinity was assessed by PXRD data using OriginPro.

**Figure 6:**
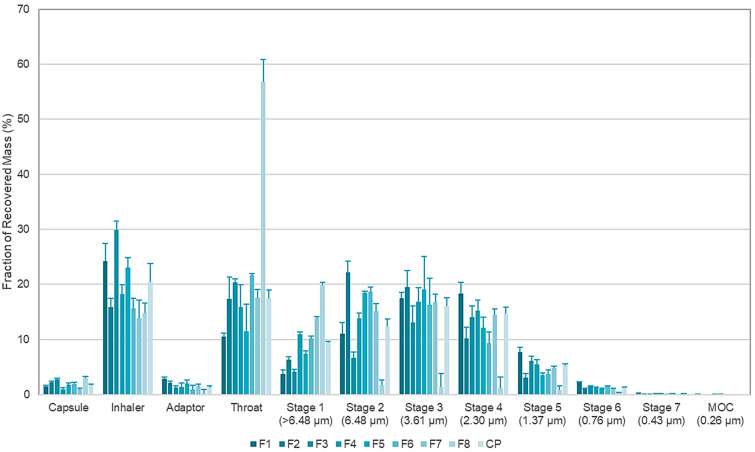
NGI deposition patterns of spray-dried FAV-THP formulations with the corresponding upper aerodynamic cutoff diameter specified. MOC: the micro-orifice collector in the NGI.

In general, for most of the tested conditions, powders with good aerosol performance were obtained (Table 4), which exhibited MMADs smaller than 5 μm except F8. The FPF of the 11 formulations varied between 5.56 (F8) to 79.3% (F1). The EF values were within 61.26 to 93.05%, which were generally regarded as acceptable in terms of powder dispersibility [65]. F1, which exhibited the smallest MMAD (2.93 μm) and a significantly higher FPF (79.3%) (*p* < 0.05), was selected as the optimal formulation with a stoichiometric ratio of 1:1.07 confirmed by the HPLC assay. Since commercially available carrier-based DPIs were reported to produce FPFs between 10% and 50% at different flow rates [78], this features the high potential of cocrystallization for developing inhalable formulations without the aid of carriers. The NGI dispersion patterns of the cocrystals indicated that 45.81% of aerosolized powders from F1 deposited on stages 3–6, where the aerodynamic diameters fell within 3.61 to 0.43 μm (Figure 6).

In the context of inhalable cocrystal development, judicious selection of coformer appeared to determine the final aerosol performance of the product. Alhalaweh et al. assessed the aerosol performance of different inhalable THP cocrystals, with urea (URE), saccharin (SAC), and nicotinamide (NIC) prepared by spray drying, in comparison to that of the spray-dried raw drug [63]. Under similar processing conditions of spray drying, THP-NIC (16.5%) had a higher FPF than THP (13.2%), whereas the formation of THP-URE (10.4%) and THP-SAC (5.8%) cocrystals deteriorated the overall aerosol performance. These observations might be linked to the varied surface chemistries and dispersive surface energies inherent to different cocrystal systems, which is a result of a change in solid form and crystal habit of a material through cocrystallization. In our study, results suggested that spray-dried THP exhibited a superior MMAD and FPF compared with FAV, while the optimized carrier-free cocrystal formulation outperformed both drug-alone formulations (Table 5). Spray-dried FAV had a low FPF of 26.69% plausibly due to its stickiness nature, which failed to meet the FPF requirement (i.e., >30%) defined in the DoE. Interestingly, cocrystallization of FAV with THP synergistically improved overall aerosolization behavior, such that the FPF_FAV_ substantially increased from 26.69 to 79.30%. The synergy created under appropriate processing conditions of spray drying might be attributed to the formation of intermolecular hydrogen bonding between FAV and THP, as well as dimeric interactions of FAV-FAV and THP-THP based on the crystal structure. This marks the promise of applying the cocrystallization with spray drying to tame the aerosolization of FAV. Thus, THP herein behaves as a potential add-on therapy in influenza patients with existing chronic lung diseases and benefits a more effective pulmonary drug delivery, providing complementary clinical advantages.

**Figure 7:**
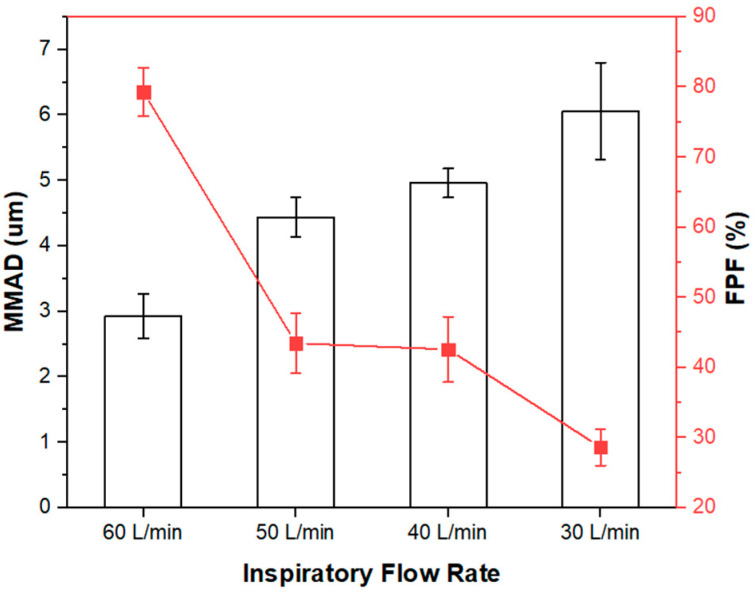
In vitro aerosolization performance of the spray-dried F1 at different inspiratory flow rates.

Although the majority of patients with COPD can achieve an inspiratory flow rate of 60 L/min [79], concurrent pulmonary viral infections of SARS-CoV-2 may worsen the degree of lung function impairment, such that sufficient inspiratory flow and turbulence may not be generated to disperse the powders. Consequently, reduced pressure drops requiring less inspiratory effort were further investigated in the NGI experiments. Figure 7 revealed that the optimized F1 formulation displayed flow-dependent aerosolization. Satisfactory aerosolization for deep lung delivery was maintained with optimal respirable particle size range and FPF of over 40%, even when the inspiratory flow rate was reduced to 40 L/min to achieve 0.7 kPa pressure drop. A FPF of 40% is regarded as reasonably acceptable aerosol performance as reported in literature [64,65,80,81,82], suggesting the potential utility of the FAV-THP cocrystal DPI formulation in patients with concomitant mild-to-moderate pulmonary viral infections (e.g., SARS-CoV-2) and COPD.

#### 3.3.2. Identification of Influential Factors for the Critical Quality Attributes in the Spray Drying Process

##### Mass Median Aerodynamic Diameter (MMAD)

**Figure 8:**
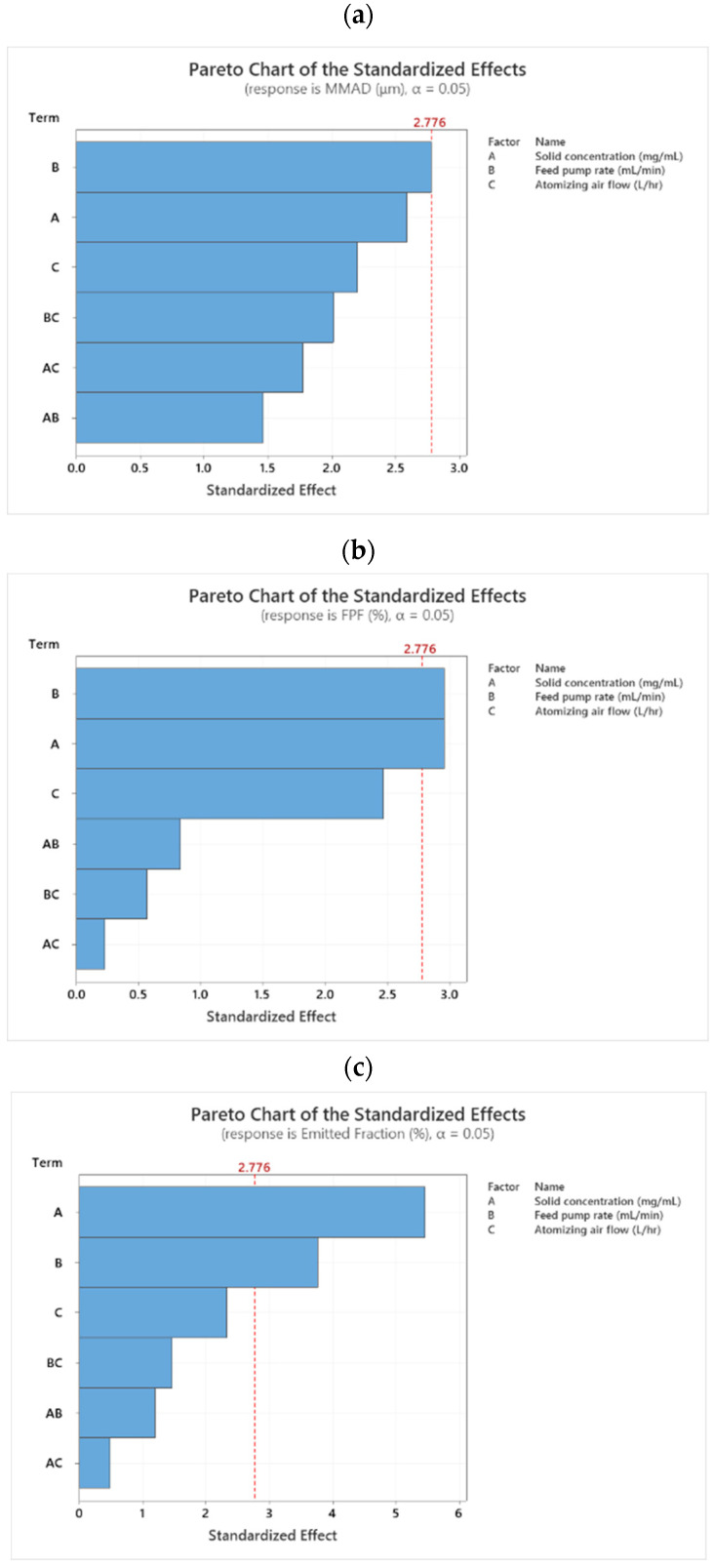

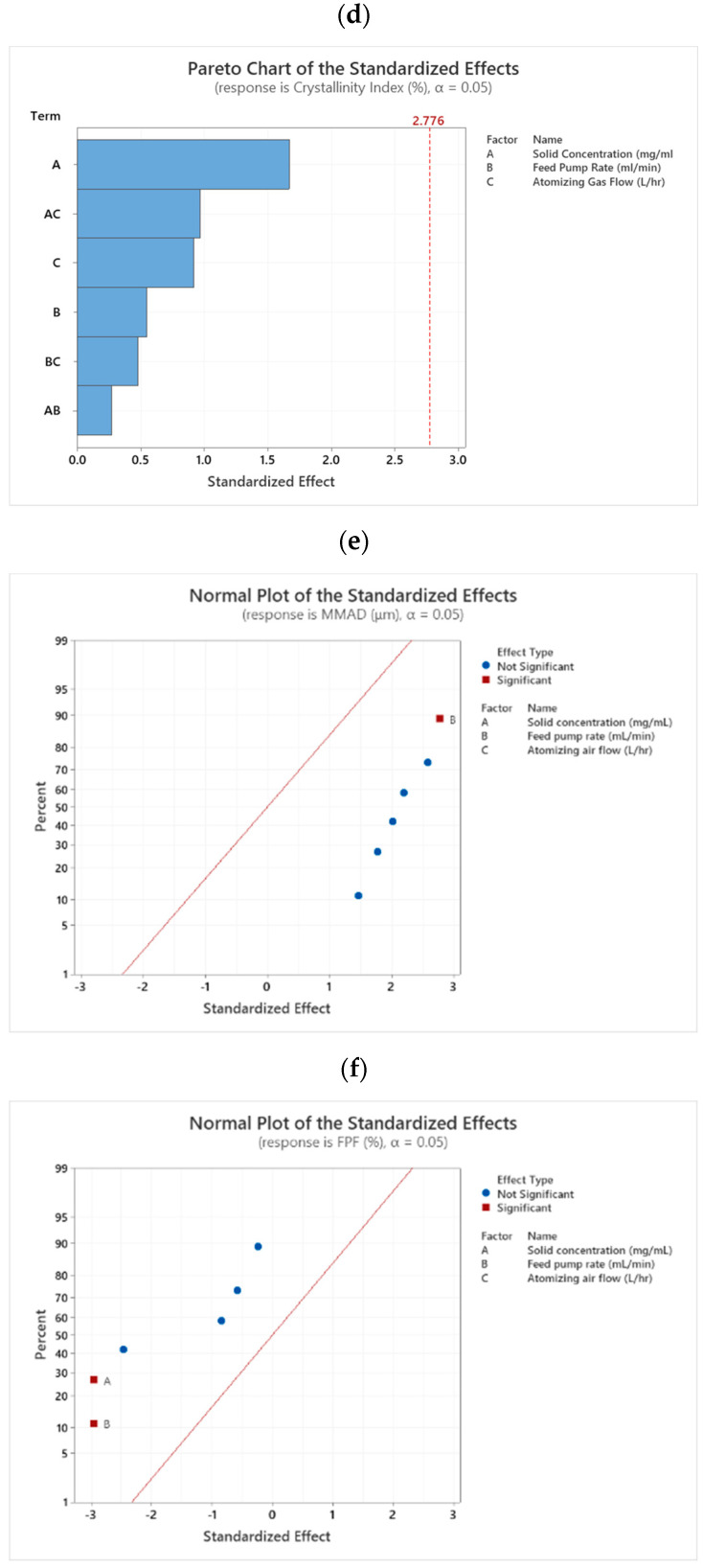

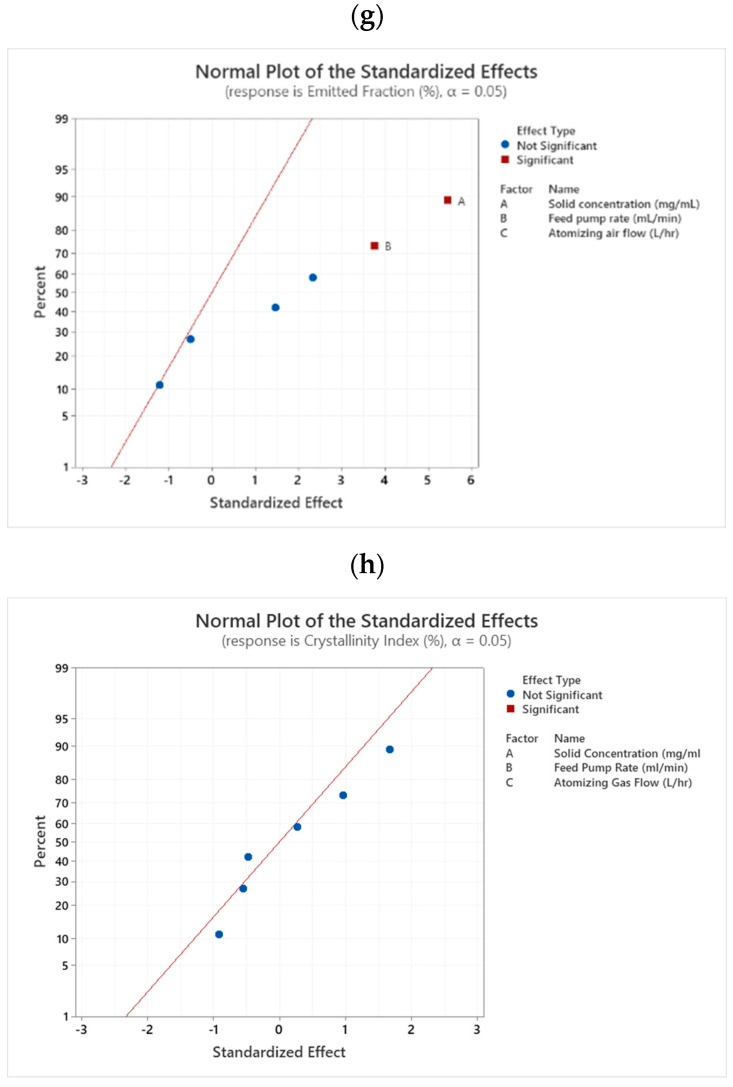
Pareto charts illustrate the standardized effect of the independent variables (A: Solid concentration, B: Feed pump rate, C: Atomizing gas flow) and their interactions on MMAD (**a**), FPF (**b**), EF (**c**), and CI (**d**) of the inhalable cocrystal formulation. The factors that cross the vertical red reference line indicate that their effects are statistically significant. Normal probability plots (**e**–**h**) are used to determine the magnitude, direction and the importance of the effects of the independent variables and their interactions on MMAD (**e**), FPF (**f**), EF (**g**), and CI (**h**). Effects further from 0 are more statistically significant. The Pareto charts and normal probability plots both indicate that the feed pump rate is a significant parameter affecting the MMAD; the solid concentration and feed pump rate are the significant parameters affecting the FPF and EF; and the CI is not significantly affected by any of the studied processing parameters.

The aerodynamic particle diameter, which is defined as the diameter of a sphere of unit density, determines the mechanisms of particle deposition, dissolution, and clearance in the respiratory system [83,84]. Effects of the total solute concentration, feed pump rate, and atomizing air flow on the MMAD of FAV-THP cocrystal powders under a constant inlet temperature are depicted in Figure 8a. The Pareto chart demonstrated that the feed pump rate is a critical factor that significantly influences the MMAD_FAV-THP_ from 1.5 mL/min to 4.5 mL/min (*p* = 0.05). The MMAD_FAV-THP_ was ranked in the following order: F1 ≈ F3 < F2 ≈ F4 ≈ F5 ≈ CPs < F6 ≈ F7 < F8. Under the same atomizing gas flow, an increased feed pump rate apparently led to enlarged FAV-THP particles due to lower atomization energies [85,86]. For example, the experimental condition for F5 resulted in an MMAD of 3.92 µm in comparison to an MMAD of 2.93 µm for F1 under the same condition whereas tripling the feed pump rate. However, it is worth noting that the effect dominated by feed pump rate seemed to be augmented in a cocrystal system when total solute concentration and atomizing airflow were simultaneously increased (MMAD_F8_ = 10.58 µm). Figure 6 illustrates that a significant fraction of the powders from F8 were trapped in the throat (56.85%), which signifies a high degree of particle agglomeration in the aerosolized state, as manifested in the large MMAD of 10.58 μm and an FPF value as low as 5.56%. This implied that the drug particles were not properly dispersed into individual particles.

Such formulation with a high EF of 93.05% but low FPF could potentially elevate the risk of adverse effects induced by an increase in unintended systemic exposure to FAV and THP [65]. Albeit the Pareto chart did not suggest total solute concentration alone as a significant factor, a smaller MMAD_FAV-THP_ was generally obtained when the total solute concentration was reduced, which is in line with our previous finding regarding the development of an inhalable itraconazole-suberic acid cocrystal formulation [64]. The resulting larger mean MMAD is attributed to the increased solute content in each atomized droplet, leading to an increased feed viscosity and enlarged primary particle size [87]. This positive effect could also result from a stronger propensity for particle agglomeration at a high degree of local supersaturation [76,88,89]. A low solute concentration imparts long interparticle distance with diffused nuclei, thereby minimizing agglomeration, which is favorable to pulmonary drug delivery. Further analysis of how the MMAD was influenced within the design space was performed by constructing 2D Contour plots and 3D surface plots, holding the atomizing gas flow at the highest level (Figures S6A and S7A). The blue color represents areas within the design space where the defined limits are met. The graphs indicated that the MMAD showed a proportional increase with the solute concentration and the feed pump rate.

##### Fine Particle Fraction (FPF) and Emitted Fraction (EF)

Apart from the aerodynamic diameter of primary particles, the dispersibility of the particles has a pronounced effect on determining the overall particle size distribution and deposition during inhalation [90,91]. Evaluation of the FPF, which represents the mass fraction of drug (with respect to the ED) with a particle size below 5 µm, is thus essential. The factors governing the FPF generated by a DPI are intricated and intercorrelated [92]. For the case of spray-dried FAV-THP cocrystal powders, Figure 8b,c revealed that the FPF and EF were significantly affected by both total solute concentration (FPF: *p* = 0.042; EF: *p* = 0.005) and feed pump rate (FPF: *p* = 0.042; EF: *p* = 0.02). As expected, factors influencing the FPF were largely the reciprocal of those influencing the MMAD, in accordance with the observations in the literature [93]. For example, an approximate 35% decrease in the FPF of cocrystal powders was detected by increasing the solute concentration from 3 mg/mL (F1) to 9 mg/mL (F2) when keeping other parameters as constants. Similarly, a 32% decrease in the FPF of cocrystal powders was seen when solely increasing the feed pump rate from 1.5 mL/min (F1) to 4.5 mL/min (F5). The changes in particle shape might explain the strong negative correlations and surface roughness may affect the FPF. Higher solute concentrations and feed rates lead to coarser particle surfaces consisting of larger and irregular-shaped crystals, as indicated by the SEM images (Figure S8) [77,94,95]. The effects of the two significant parameters on FPF and EF are graphically represented as 2D Contour plots and 3D surface plots (Figures S6B,C and S7B,C).

##### Crystallinity

Unintentional amorphization of APIs in the presence of excipient is one of the undesirable issues encountered during the manufacturing process of inhalable powders. Many excipients, especially sugars bearing high molecular weight with a high T_g_ and low moisture content, undergo phase transformation to a thermodynamically unstable amorphous state upon spray drying [86]. Hygroscopic amorphous powders with allied higher surface free energy are prone to agglomeration. Consequently, deterioration in aerosol performance is encountered from increased interparticulate capillary forces [86,96,97]. To this end, it is of great interest to explore whether our carrier-free inhalable FAV-THP powder formulations would show any sign of co-amorphization under different spray drying processing conditions. On the basis of the PXRD and DSC data (Figure S9), spray drying was found to be robust in producing crystalline FAV-THP cocrystal powders for inhalation. All spray-dried formulations were apparently phase pure with a sole melting temperature at around 195° and absence of a detectable glass transition and recrystallization. The five characteristic diffraction peaks of the cocrystal remained with notable intensity.

Crystallinity index (CI) was used as a quantitative indicator to compare the crystallinity of different FAV-THP formulations. The CI was calculated from the raw PXRD data, which is the ratio of the area of all crystalline peaks to the total area comprising the crystalline and amorphous content [67,98,99,100]. High-angle Bragg peaks (>20°) were excluded in this analysis as poorer counting statistics could be resulted from high-angle data, due to the combined effects of a decrease in the scattering coefficient with increasing sin θ/λ, Lorentz–polarization factor and thermal vibrations [101,102]. CIs of the 11 formulations ranged from 57 to 75% (Table 4). This suggests certain of amorphous contents are involved in some formulations. The main concern of the formulation being partly amorphous could be the resulting deterioration of stability, which may hamper the therapeutic effect of the cocrystal. However, based on the stability data mentioned earlier, no detrimental stability issue of the optimized formulation under stressed temperature and humidity conditions was found. Thus, the minor amorphous content present here is not expected to be a significant concern during the later drug development.

The Pareto chart (Figure 8d) indicated that neither the total solute concentration, feed pump rate, nor atomizing gas flow is a significant factor influencing the crystallinity of the products under our tested conditions. The small differences in this measured property would be beneficial for producing consistent cocrystal powders with high crystallinity during the stage of technology transfer. The result also implies crystallinity is more dependent on the intrinsic properties of cocrystal formers and the tendency of cocrystal formation than the processing parameters. For example, spray drying of another drug-drug combination of THP and budesonide by Leng et al. produced co-amorphous powders [103]. Spray drying of metastable cocrystal systems such as itraconazole-suberic acid led to amorphization or formation of physical mixture with low crystallinity regardless of the processing parameters [53,64]. Although the higher free energy facilitates the rearrangement of disordered co-amorphous molecules into ordered crystalline phase, surmounting the energy barrier contributed by the entropy change (ΔS) is the prerequisite for the nucleation of crystallites [104]. Solidification of the cocrystal former liquids into the kinetically stable cocrystal solid confers a larger ΔS than that into the thermodynamically stable cocrystal solid, rendering the kinetically stable system to retain in co-amorphous state upon drying.

### 3.4. Characterization of the Optimized Spray-Dried Cocrystal Particles

#### 3.4.1. Morphology

**Figure 9:**
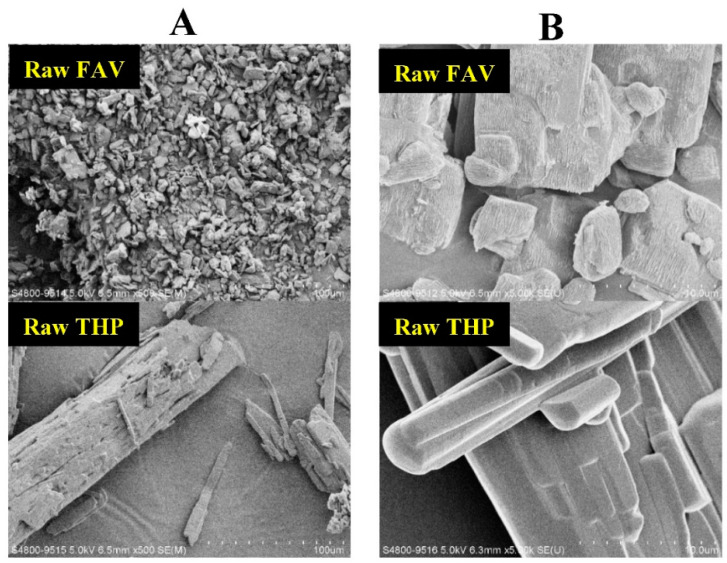

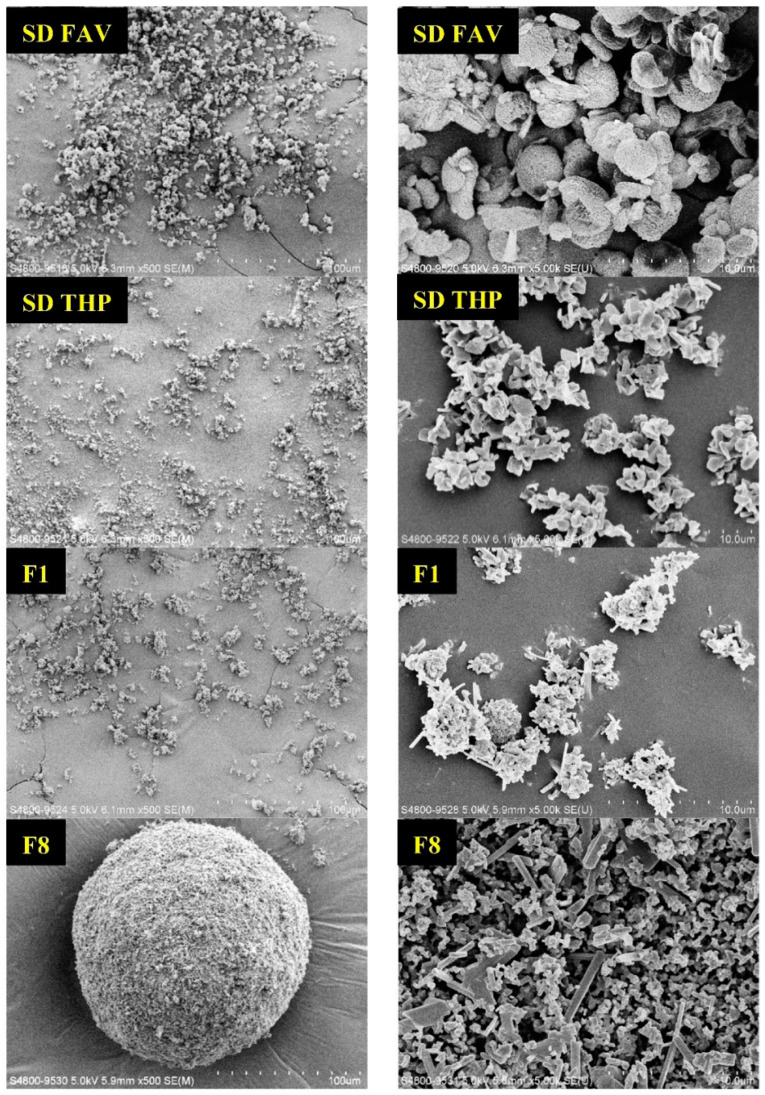
Scanning electron micrographs of raw cocrystal formers, spray-dried cocrystal formers, and spray-dried FAV-THP cocrystal powders (F1 vs. F8 formulations) at 500× magnification (Panel **A**) and 5000× magnification (Panel **B**).

The morphology of the spray-dried FAV-THP cocrystal powders in comparison to the cocrystal formers was examined by SEM (Figure 9). The FAV alone spray-dried powders were generally dimpled spheres, whereas the spray-dried THP powders displayed flake-shaped structures with smoother surfaces. At high magnification, no striking difference was observed among the spray-dried cocrystal powder formulations and their morphology resembles that of the spray-dried THP powders, except that occasionally a few elongated rod-like particles were present (Figure S8). The change of surface morphology and roughness of spray-dried FAV induced by cocrystallization is expected to exert a positive impact on the overall aerosol performance. The cocrystal powders exhibited porous structure and formed clusters with different degrees of agglomeration. It was evident that samples prepared at the high level CPPs were more prone to agglomeration, while those prepared at lower feed pump rate and solute concentration appeared as discrete units (e.g., F1 and F3). The SEM image suggests that the smaller particle sizes of F1 could be due to the less aggregating structures (Figure 9). This implies when pressure was applied during powder dispersion, F1 can easily undergo deagglomeration which resulted in a higher FPF over other formulations in the NGI experiments (Table 4). The surface area of the particles was augmented by deagglomeration, leading to enhanced interaction between the particles, capsule and inhaler during dispersion due to the electrostatic charge [65]. This could plausibly explain why more than 20% drug of F1 was deposited inside the capsule and inhaler, thus showing a relatively lower EF (Figure 6). On the other hand, the SEM image of F8 at low magnification revealed a formation of an extremely dense network such that an abundance of micronized particles fused with each other to form a large sphere with a diameter >100 μm (Figure 9). The volumetric size distribution of spray-dried cocrystal powders was measured by laser diffractometry (Table 4 and Table 5). When the powders were aerosolized by Breezhaler^®^ at 60 L/min, the D_50_ varied from 3.83 μm (F1) to 6.92 μm (F8) across all formulations. This further substantiates that the large sphere of F8 observed under the SEM was more likely due to a high degree of particle agglomeration instead of an enlarged primary particle size.

#### 3.4.2. Dissolution Performance

**Figure 10:**
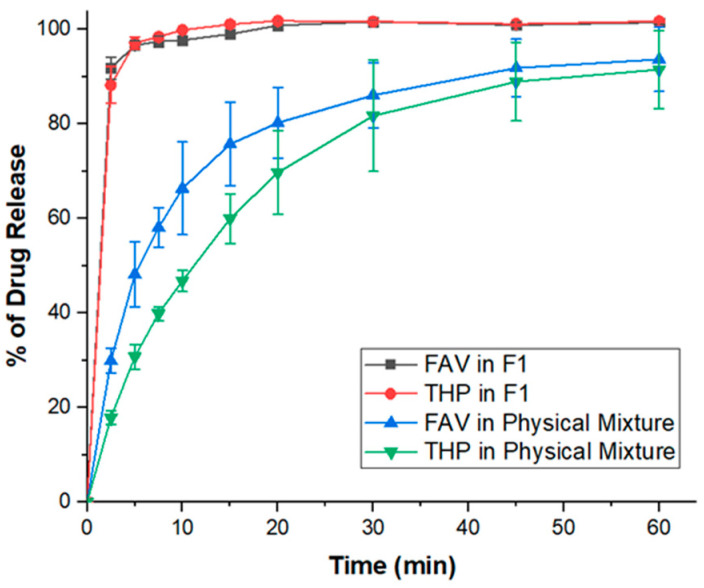
In vitro dissolution profile of FAV and THP in the optimized spray-dried cocrystal formulation F1 with aerodynamic diameters <5 μm (*n* = 3).

The dissolution performance in the lungs could be a rate-limiting step for the overall absorption of inhalable pharmaceuticals intended for the treatment of chronic lung diseases [105]. Slow dissolving drugs may be cleared either by the mucociliary escalator in the upper airway to the esophagus where they are swallowed or by macrophage sequestration in the alveolar region [106]. To this end, the dissolution profile of the optimized spray-dried FAV-THP cocrystal formulation (F1) was compared with its parent constituents in PBS buffer. There is no regulatory requirement or pharmacopeial protocol available for testing the dissolution behavior of inhalable formulations. This study adopted a dissolution method reported by Liao et al. to mimic the conditions in lungs [68]. One of the challenges in developing a suitable dissolution method for DPI formulation is to control the particle size effect. Unlike dissolution test for oral dosage form where sifting is required to control the particle size. A Fast Screening Impactor is usually used for collecting the respirable size dry powder particles with an MMAD < 5 μm for subsequent dissolution test, as reported in the literature [64,65,68]. As no inhalable formulation of FAV/THP is currently in the market, while dose conversion between oral and inhalable FAV/THP is ambiguous where no universal protocol is available, a 10 mg of powder was used for dispersion to collect the fine particles for comparison purpose. The FPF of spray-dried FAV-THP powders using FSI, assayed by HPLC, was 79.5%. The dissolution test for the unformulated FAV and THP was not conducted with fine particle dose, as their particle size was too large to be collected by the FSI. Raw FAV exhibited a faster dissolution rate than raw THP in the physical mixture (Figure 10), of which the drug release reached 29.9 ± 2.6% and 17.3 ± 1.5% at 2.5 min in simulated lung fluid (SNF), respectively. In contrast, the spray-dried F1 powders conferred a superior dissolution enhancement over the physical mixture, as it dissolved rapidly and was completely released within 20 min. The observed dissolution improvement brought by cocrystallization could be attributed to a combination effect of solubility advantage (Table S3) and particle size reduction. Table S3 showed that both FAV and THP exhibit poor aqueous solubility. Cocrystallization of FAV with THP significantly increased the solubility of FAV (*p* = 0.014), whereas reduced the solubility of THP. As mentioned earlier, SEM image suggested that the spray-dried F1 powders can easily undergo deagglomeration during dispersion, which greatly increased the specific surface area in contact with SNF and hence, leading to improved dissolution performance.

#### 3.4.3. Cytotoxicity

**Figure 11:**
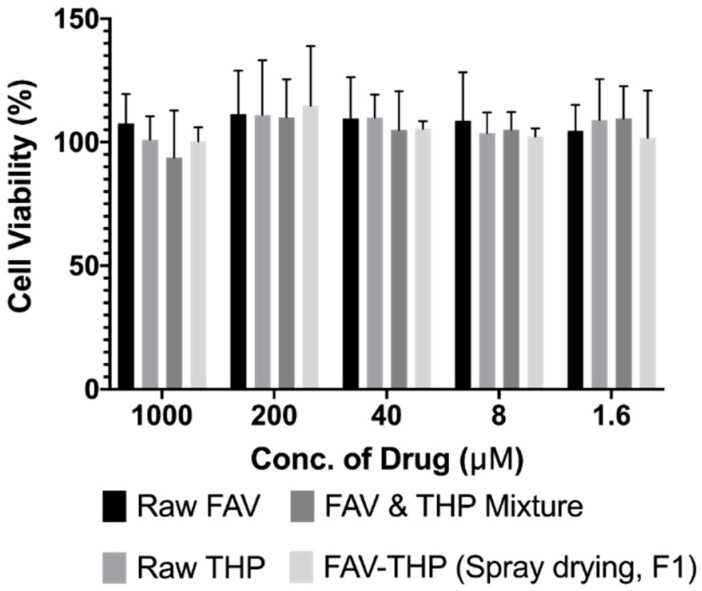
Cell viability of the raw drugs, the physical mixture and the spray-dried F1 at concentrations from 1.6 to 1000 µM.

The cytotoxicity of the optimized spray-dried cocrystal particles was evaluated using the MTT cell viability assay, compared with the raw FAV and THP as well as the FAV-THP physical mixture. A549 cells were selected because it is the one of the most studied human lung epithelial cell lines. In the concentration range of 1.6–1000 µM, the cell viability of all the groups was around 100% and no significant difference was found (*p* > 0.05, Figure 11). Therefore, no cytotoxicity was observed in the A549 cells, indicating a favorable in vitro safety of the formulation at concentrations from 1.6 to 1000 µM. The highest concentration of this MTT assay was chosen according to a previous open-label observational study evaluating the efficacy of favipiravir to treat Ebola, where the highest plasma concentration of favipiravir observed in patients was 173.2 µg/mL (~1100 µM) [107]. Oestereich et al. also investigated the efficacy of favipiravir against Zaire Ebola virus in vitro using concentrations up to 1000 µM [108]. As the volume of lung fluid is limited, around 0.37 mL/kg body weight, it is easy to achieve such concentration in the lung through inhalation of the cocrystals. For a human with a body weight of 60 kg, around 9.5 mg of F1 can achieve a concentration of 1000 µM in the lung. In the future in vivo study, smaller doses will be used to ensure the safety of animals. It should be noted that the utility of drug-drug cocrystal in medicine is generally restricted by the inflexible dosage regimen, considered as one of the major challenges in the formulation development [54]. Excessive amount of either cocrystal formers appears inevitable, which has been seen in many reported systems and may pose toxicity concern. The stoichiometric ratio of the FAV-THP cocrystal was found as 1:1. Although the dose of cocrystal formers may not be in agreement with its recommended therapeutic dose for the indication, it is worth noting that cocrystallization imparts potential enhancement in bioavailability. In addition, reformulating both oral FAV and THP as inhalable cocrystal dosage form changes the route of administration and allows the drugs to directly target the lungs. Dose reduction is possible for achieving an equivalent therapeutic efficacy to the existing formulation and therefore, mitigating toxicity. In this situation, adequate dose-finding and in vivo pharmacokinetic studies must be conducted in the later stage of drug development to establish the safety profile of the inhalable formulation, whereas out of the scope of the present study.

### 3.5. Significance

The integrated cocrystallization with spray drying process represents a novel particle engineering strategy for the development of carrier-free inhalable DPI. The multi-drug cocrystal dry powders could possess synergistic improvement of pharmaceutical properties, where the flexible design of coformers can facilitate an advanced delivery of personalized medicine to individual patient with acute/chronic respiratory infections. Micronized particles in conventional DPI possess cohesiveness and strong aggregation propensity. To improve flowability and dispersion of drug particles during emission, blending with inactive carrier (e.g., lactose, mannitol) was conventionally deemed critical for product delivery. The U.S. Food and Drug Administration (FDA) scrutinizes the CQAs associated with excipients including assay, toxicity, particle morphology, flow properties, amorphous and moisture contents, etc., stressing their potential impacts on final product quality for pulmonary delivery [109]. From a regulatory perspective, it should be highlighted only a handful of excipients have been approved for pulmonary drug delivery [45]. In this study, we have demonstrated a robust single-step method for manufacturing a carrier-free antiviral drug-drug cocrystal DPI with good aerosol performance and acceptable in vitro cytotoxicity profile using spray drying, negating any concerns about the safety and tolerability of additional excipients in the formulation. In light of the capability to simultaneously improve a series of physicochemical properties, multi-drug cocrystallization confers intrinsic promise in minimizing the use of excipient, allowing high dose delivery. With careful selection of drug coformers, it offers opportunities in achieving personalized combination therapy for individual patients. This is of paramount importance in the modern society considering polypharmacy and multicomorbidity are very common phenomena in clinics. However, unlike the drug-carrier systems of which the ratio of drug substance to excipient can be flexibly adjusted, the fixed stoichiometric ratio of drug-drug cocrystal in DPI could be a hinderance for pharmaceutical development. Future investigation is warranted to establish the in vivo pharmacokinetics and efficacy profiles of the inhalable FAV-THP cocrystal.

## 4. Conclusions

The use of QbD allows an expedited development of a carrier-free inhalable FAV-THP cocrystal powder medication by an integrated cocrystallization and spray drying technology in response to a public health emergency. The novelty of this work lies in demonstrating that spray drying is a robust particle engineering technique for producing cocrystal as an inhalable FAV-THP dry powder formulation. The single cocrystal of FAV-THP was found in a 1:1 stoichiometry, and the structure exhibited an FAV-FAV to THP-THP assembly. PXRD analysis demonstrated that the spray-dried products remained in a highly crystalline state within the design space. Without the aid of excipient, the optimized FAV-THP cocrystal formulation exhibited desired properties for pulmonary drug delivery, with enhanced dissolution rate and favorable in vitro cytotoxicity profile. It showed an MMAD of 2.93 μm and an FPF of 79.3% when dispersed at 60 L/min, which outperformed both drug-alone formulations. An acceptable respirable particle size range and FPF of over 40% can be achieved when further reducing the inspiratory flow rate to 40 L/min, and thus it can be used as a potential inhalable SARS-CoV-2 treatment for patients with underlying COPD/asthma. Further study on the in vivo evaluation of the inhalable formulations is warranted to facilitate clinical translation.

## Figures and Tables

**Table 1:** Design of the two-level three-factor full factorial DOE to study the spray dried FAV-THP cocrystal powder.

	Independent Processing Variables (CPPs)		Levels	
		Low (−1)	Mid-Point (0)	High (+1)
X_1_	Total solute concentration (mg/mL)	3	6	9
X_2_	Feed pump rate (mL/min)	1.5	3	4.5
X_3_	Atomizing air flow (L/h)	357	536	742
	**Responses:** **Dependent Variables (CQAs)**	**Goal**	**Acceptable Range**	
Y_1_	Mass median aerodynamic diameter (MMAD, μm)	3	1–5	
Y_2_	Fine particle fraction (FPF, %)	Maximize	≥30	
Y_3_	Emitted fraction (EF, %)	Maximize	≥60	
Y_4_	Crystallinity Index (CI, %)	Maximize	≥50	

**Table 2:** Overview of the combinations of the material attribute and process parameters adopted.

Formulation	Total Solute Concentration (mg/mL)	Feed Pump Rate (mL/min)	Atomizing Air Flow (L/h)
F1	3	1.5	357
F2	9	1.5	357
F3	3	1.5	742
F4	9	1.5	742
F5	3	4.5	357
F6	9	4.5	357
F7	3	4.5	742
F8	9	4.5	742
CP1	6	3	536
CP2	6	3	536
CP3	6	3	536

**Table 3:** Overview of the combinations of the material attribute and process parameters adopted.

Peak Assignment	FAV (cm^−1^)	THP (cm^−1^)	FAV-THP by Rotary Evaporation (cm^−1^)	FAV-THP by Spray Drying (cm^−1^)
*ν* (NH_2_)	3346~3269 (broad)	3120	3359~3207 (broad)	3357~3207 (broad)
*ν* (OH)	3207	–	3104	3105
*ν* (C=O)	1659	1663	1642	1643
δ (NH_2_)	1600	1561	1600	1599
*ν* (CN)_amide_	1394	–	1395	1394

**Table 4:** Aerodynamic size distribution (MMAD, GSD, FPF, and EF), crystallinity index (CI), and volumetric particle size distribution (D10, D50, D90, SPAN) of spray-dried FAV-THP formulations under different processing parameters. N = 3 with standard deviations shown in brackets.

	MMAD (μm)	GSD (μm)	FPF (%)	EF (%)	D_10_ (μm)	D_50_ (μm)	D_90_ (μm)	SPAN	CI (%)
F1	2.93 (0.34)	1.91 (0.11)	79.30 (3.44)	61.26 (2.59)	1.16 (0.05)	3.83 (0.03)	10.64 (0.10)	2.48 (0.02)	69.13
F2	3.96 (0.09)	1.98 (0.06)	51.68 (7.02)	82.14 (5.31)	1.84 (0.13)	5.03 (0.14)	10.01 (0.19)	1.62 (0.04)	74.54
F3	3.04 (0.16)	1.93 (0.11)	53.76 (3.70)	66.63 (6.44)	1.65 (0.18)	4.20 (0.24)	9.93 (0.23)	1.97 (0.10)	66.36
F4	3.87 (0.26)	2.02 (0.029)	51.01 (5.66)	80.24 (2.74)	1.25 (0.04)	4.46 (0.08)	14.20 (0.12)	2.90 (0.04)	72.95
F5	3.92 (0.28)	1.99 (0.06)	54.10 (1.23)	72.69 (3.01)	1.51 (0.3)	4.90 (0.36)	12.36 (0.45)	2.21 (0.12)	71.21
F6	4.23 (0.07)	2.11 (0.05)	42.64 (3.51)	82.61 (2.66)	1.66 (0.21)	5.40 (0.26)	10.70 (0.29)	1.67 (0.06)	71.83
F7	4.22 (0.12)	2.05 (0.02)	48.52 (5.81)	80.90 (4.50)	1.51 (0.07)	5.79 (0.08)	17.88 (0.12)	2.83 (0.04)	57.22
F8	10.58 (0.64)	2.48 (0.31)	5.56 (2.45)	93.05 (10.68)	1.36 (0.12)	6.92 (0.170	34.74 (0.36)	4.82 (0.08)	73.25
CP1	3.81 (0.12)	2.03 (0.19)	49.25 (4.48)	69.66 (5.33)	1.45 (0.40)	4.77 (0.39)	12.98 (0.06)	2.42 (0.23)	63.38
CP2	3.68 (0.45)	2.01 (0.37)	53.54 (5.79)	77.19 (4.39)	1.40 (0.13)	4.86 (0.06)	14.47 (0.15)	2.69 (0.03)	61.89
CP3	3.74 (0.22)	2.00 (0.18)	54.12 (4.01)	76.72 (2.23)	1.49 (0.22)	4.91 (0.18)	12.92 (0.09)	2.33 (0.10)	61.59

**Table 5:** Aerodynamic and volumetric size distribution of spray-dried pure FAV, pure THP, and optimized FAV-THP formulation (F1) under identical experimental conditions. N = 3 with standard deviations shown in brackets.

	MMAD (μm)	GSD (μm)	FPF (%)	EF (%)	D_10_ (μm)	D_50_ (μm)	D_90_ (μm)	SPAN
FAV	4.91 (0.42)	2.20 (0.21)	26.69 (5.81)	79.07 (7.51)	1.99 (0.24)	6.15 (0.26)	12.92 (0.6)	1.78 (0.02)
THP	3.66 (0.19)	1.95 (0.09)	46.71 (2.36)	90.95 (4.78)	1.56 (0.04)	4.03 (0.10)	8.91 (0.02)	1.82 (0.01)
F1	2.93 (0.34)	1.91 (0.11)	79.30 (3.44)	61.26 (2.59)	1.16 (0.05)	3.83 (0.03)	10.64 (0.10)	2.48 (0.02)

## Data Availability

All data available are reported in the article.

## Supplementary Materials

The following are available online at https://www.mdpi.com/article/10.3390/pharmaceutics14020300/s1, Figure S1: Chemical structures of the two APIs (a) Favipiravir and (b) Theophylline, Figure S2: DSC thermograms of the FAV-THP cocrystal produced by rotary evaporation and spray drying after stability test, Figure S3: Water sorption isotherms at 25 °C of the FAV-THP cocrystals produced by different methods, Figure S4: Temperature-composition phase diagram of the FAV-THP cocrystal system, Figure S5: TGA profiles of the FAV-THP cocrystal system, Figure S6: 2D contour plots for the (A) MMAD, (B) FPF, (C) EF, and (D) CI as projections of the total solute concentration (x-axis) and the feed pump rate (y-axis), holding a high level atomizing gas flow, Figure S7: 3D surface plots for the (A) MMAD, (B) FPF, (C) EF, and (D) CI at high level atomizing gas flow, Figure S8: Scanning electron micrographs of different spray-dried cocrystal dry powder formulations at 5000× magnification, Figure S9: PXRD (a) and DSC (b) profiles of different spray-dried FAV-THP formulations, Table S1: Experimental outlet temperatures resulted in the DOE, Table S2: The powder dispersion was performed at various flow rates to obtain 4 L passing air drawn into the next generation impactor (NGI) with designated pressure drops, Table S3: Equilibrium solubilities of coformers and cocrystal at 20 °C, Table S4: Crystal data and structure refinement for THP:FAV, Table S5: Atomic coordinates (× 10^4^) and equivalent isotropic displacement parameters (Å^2^ × 10^3^) for THP:FAV, Table S6: Bond lengths [Å] and angles [°] for THP:FAV, Table S7: Anisotropic displacement parameters (Å^2^ × 10^3^) for THP:FAV, Table S8: Hydrogen coordinates (×10^4^) and isotropic displacement parameters (Å^2^ × 10^3^) for THP:FAV, Table S9: Hydrogen bonds with H..A < r(A) + 2.000 Angstroms and <DHA > 110 deg for THP:FAV.
