# Supplementary material for: Rational Development of a Carrier-Free Dry Powder Inhalation Formulation for Respiratory Viral Infections via Quality by Design: A Drug-Drug Cocrystal of Favipiravir and Theophylline
Source: Pharmaceutics. 2022 Jan 27;14(2):300. doi: 10.3390/pharmaceutics14020300 (PMC8876093; doi:10.3390/pharmaceutics14020300)
Supplement: Supplementary file 1 [file pharmaceutics-14-00300-s001.zip › pharmaceutics-1526841-supplementary.pdf]

pharmaceutics

Supplementary Materials: Rational Development of a Carrier-Free Dry Powder Inhalation Formulation for Respiratory Viral Infections via Quality by Design: A Drug-Drug Cocrystal of Favipiravir and Theophylline

Si Nga Wong, Jingwen Weng, Ignatius Chun Wai Ip, Ruipeng Chen, Richard Lakerveld, Richard Telford, Nicholas Blagden, Ian J. Scowen and Shing Fung Chow

Table S1. Experimental outlet temperatures resulted in the DOE.

	F1	F2	F3	F4	F5	F6	F7	F8	CPs
**Outlet Temperature (°C)**	56	58	54	53	52	54	49	48	54

Table S2. The powder dispersion was performed at various flow rates to obtain 4 L passing air drawn into the next generation impactor (NGI) with designated pressure drops.

**Flow rate (L/min)**	**Pressure difference (kPa)**	**Time required (s)**
30	0.4	8.0
40	0.7	6.0
50	1.1	4.8
60	1.4	4.0

Table S3. Equilibrium solubilities of coformers and cocrystal at 20°C (n=3).

**Chemical Species**	**Aqueous Solubility (mg/mL) (± SD)**
Raw FAV	2.29 ± 0.34
Raw THP	7.14 ± 0.47
FAV-THP	FAV: 3.11 ± 0.03<br>THP: 3.42 ± 0.43

Table S4. Crystal data and structure refinement for THP:FAV.

**Identification code**	**cu_IS_HKU_4FAV_THP_SEBatch2_0m**	
Empirical formula	C12 H12 F N7 O4	
Formula weight	337.29	
Temperature	173(2) K	
Wavelength	1.54178 Å	
Crystal system	Monoclinic	
Space group	$P2_1/n$	
Unit cell dimensions	a = 13.3490(6) Å	= 90°.
	b = 7.3654(3) Å	= 107.7362(14)°.
	c = 14.7643(6) Å	= 90°.
Volume	1382.64(10) Å3	
Z	4	
Density (calculated)	1.620 Mg/m3	
Absorption coefficient	1.161 mm-1	
F(000)	696	
Crystal size	0.296 x 0.220 x 0.076 mm3	
Theta range for data collection	3.913 to 72.167°.	
Index ranges	-15<=h<=16, -9<=k<=9, -18<=l<=18	
Reflections collected	41916	
Independent reflections	2730 [R(int) = 0.0402]	
Completeness to theta = 67.679°	99.90%	
Absorption correction	Semi-empirical from equivalents	
Max. and min. transmission	0.7536 and 0.6120	
Refinement method	Full-matrix least-squares on F2	
Data / restraints / parameters	2730 / 0 / 269	
Goodness-of-fit on F2	1.033	
Final R indices [I>2sigma(I)]	R1 = 0.0356, wR2 = 0.0962	
R indices (all data)	R1 = 0.0368, wR2 = 0.0974	
Extinction coefficient	0.0016(3)	
Largest diff. peak and hole	0.314 and -0.174 e.Å-3	

Table S5. Atomic coordinates (x 10^4) and equivalent isotropic displacement parameters ($Å^2$x 10^3) for THP:FAV. U(eq) is defined as one third of the trace of the orthogonalized U^{ij} tensor.

	x	y	z
C(10)	5685(1)	1279(2)	-7005(1)
O(10)	6137(1)	1101(1)	-3988(1)
N(10)	5528(1)	962(2)	-6166(1)
C(11)	7033(1)	2328(2)	-5975(1)
O(11)	9156(1)	3896(1)	-4104(1)
N(11)	6592(1)	2129(2)	-6930(1)
N(12)	7981(1)	3128(1)	-5519(1)
C(12)	6403(1)	1628(2)	-5482(1)
C(13)	6667(1)	1688(2)	-4477(1)
N(13)	7638(1)	2524(2)	-4062(1)
C(14)	8313(1)	3221(2)	-4542(1)
C(15)	8012(1)	2678(3)	-3023(1)
C(16)	8680(1)	3772(2)	-6049(1)
C(24)	6472(1)	3635(2)	353(1)
C(23)	7839(1)	2181(2)	-265(1)
C(22)	9338(1)	1012(2)	752(1)
O(22)	5960(1)	4116(1)	-474(1)
N(22)	6147(1)	3925(2)	1100(1)
C(21)	8989(1)	1550(2)	1503(1)
N(21)	8757(1)	1327(2)	-138(1)
O(21)	7266(1)	2496(2)	-1163(1)
C(20)	7501(1)	2698(2)	511(1)
N(20)	8098(1)	2361(1)	1407(1)
F(20)	9606(1)	1234(1)	2398(1)

Table S6. Bond lengths [Å] and angles [°] for THP:FAV.

C(10)-N(11)	1.3372(17)
C(10)-N(10)	1.3389(17)
C(10)-H(10)	0.950(17)
O(10)-C(13)	1.2317(15)
N(10)-C(12)	1.3806(15)
N(10)-H(10N)	0.891(18)
C(11)-N(11)	1.3609(15)
C(11)-C(12)	1.3691(17)
C(11)-N(12)	1.3724(15)
O(11)-C(14)	1.2198(15)
N(12)-C(14)	1.3763(15)
N(12)-C(16)	1.4678(15)
C(12)-C(13)	1.4180(16)
C(13)-N(13)	1.3971(16)
N(13)-C(14)	1.4018(15)
N(13)-C(15)	1.4662(16)
C(15)-H(151)	1.00(2)
C(15)-H(152)	1.03(2)
C(15)-H(153)	0.92(2)
C(16)-H(161)	0.92(3)
C(16)-H(162)	0.94(2)
C(16)-H(163)	1.00(2)
C(16)-H(164)	1.0831(14)
C(16)-H(165)	0.9932(14)
C(16)-H(166)	1.0781(14)
C(24)-O(22)	1.2555(15)
C(24)-N(22)	1.3192(16)
C(24)-C(20)	1.4906(18)
C(23)-O(21)	1.3333(17)
C(23)-N(21)	1.3393(18)
C(23)-C(20)	1.4064(18)
C(22)-N(21)	1.328(2)
C(22)-C(21)	1.3845(19)
C(22)-H(22)	0.966(17)
N(22)-H(23N)	0.898(18)
N(22)-H(22N)	0.890(18)
C(21)-N(20)	1.2996(17)
C(21)-F(20)	1.3487(16)
O(21)-H(21O)	0.93(2)
C(20)-N(20)	1.3427(16)
N(11)-C(10)-N(10)	113.50(11)

N(11)-C(10)-H(10)	123.0(10)
N(10)-C(10)-H(10)	123.5(10)
C(10)-N(10)-C(12)	106.14(10)
C(10)-N(10)-H(10N)	127.5(11)
C(12)-N(10)-H(10N)	126.3(11)
N(11)-C(11)-C(12)	111.61(11)
N(11)-C(11)-N(12)	126.70(11)
C(12)-C(11)-N(12)	121.69(11)
C(10)-N(11)-C(11)	103.34(10)
C(11)-N(12)-C(14)	119.32(10)
C(11)-N(12)-C(16)	121.24(10)
C(14)-N(12)-C(16)	119.30(10)
C(11)-C(12)-N(10)	105.40(10)
C(11)-C(12)-C(13)	123.21(11)
N(10)-C(12)-C(13)	131.37(11)
O(10)-C(13)-N(13)	121.30(11)
O(10)-C(13)-C(12)	126.77(11)
N(13)-C(13)-C(12)	111.93(10)
C(13)-N(13)-C(14)	126.43(10)
C(13)-N(13)-C(15)	117.85(10)
C(14)-N(13)-C(15)	115.70(10)
O(11)-C(14)-N(12)	121.73(11)
O(11)-C(14)-N(13)	120.88(11)
N(12)-C(14)-N(13)	117.39(10)
N(13)-C(15)-H(151)	107.5(11)
N(13)-C(15)-H(152)	108.6(13)
H(151)-C(15)-H(152)	109.4(18)
N(13)-C(15)-H(153)	107.7(13)
H(151)-C(15)-H(153)	111.6(18)
H(152)-C(15)-H(153)	111.9(19)
N(12)-C(16)-H(161)	109.6(15)
N(12)-C(16)-H(162)	110.5(13)
H(161)-C(16)-H(162)	103(2)
N(12)-C(16)-H(163)	108.3(12)
H(161)-C(16)-H(163)	111.8(19)
H(162)-C(16)-H(163)	113.9(17)
N(12)-C(16)-H(164)	108.14(10)
N(12)-C(16)-H(165)	110.58(11)
H(164)-C(16)-H(165)	116.02(12)
N(12)-C(16)-H(166)	112.38(11)
H(164)-C(16)-H(166)	105.77(11)
H(165)-C(16)-H(166)	103.89(11)
O(22)-C(24)-N(22)	123.21(12)
O(22)-C(24)-C(20)	119.05(11)

N(22)-C(24)-C(20)	117.73(11)
O(21)-C(23)-N(21)	116.32(12)
O(21)-C(23)-C(20)	122.33(13)
N(21)-C(23)-C(20)	121.34(12)
N(21)-C(22)-C(21)	120.29(13)
N(21)-C(22)-H(22)	119.0(9)
C(21)-C(22)-H(22)	120.7(9)
C(24)-N(22)-H(23N)	116.8(11)
C(24)-N(22)-H(22N)	121.0(11)
H(23N)-N(22)-H(22N)	121.5(15)
N(20)-C(21)-F(20)	116.86(12)
N(20)-C(21)-C(22)	124.42(13)
F(20)-C(21)-C(22)	118.71(12)
C(22)-N(21)-C(23)	117.02(12)
C(23)-O(21)-H(21O)	102.8(14)
N(20)-C(20)-C(23)	120.78(12)
N(20)-C(20)-C(24)	118.80(11)
C(23)-C(20)-C(24)	120.42(12)
C(21)-N(20)-C(20)	116.14(11)

Table S7. Anisotropic displacement parameters ($Å^2$x 10^3) for THP:FAV. The anisotropic displacement factor exponent takes the form: $-2 \;\; ^{2}[\, h^2 a^{*2}U^{11} + ... + 2\, h\, k\, a^{*}\, b^{*}\, U^{12} \,]$

	U^{11}	U^{22}	U^{33}	U^{23}	U^{13}	U^{12}
C(10)	22(1)	33(1)	21(1)	-1(1)	4(1)	0(1)
O(10)	26(1)	41(1)	26(1)	1(1)	12(1)	-9(1)
N(10)	16(1)	30(1)	23(1)	-1(1)	5(1)	-3(1)
C(11)	18(1)	20(1)	21(1)	1(1)	6(1)	3(1)
O(11)	23(1)	44(1)	26(1)	-3(1)	6(1)	-12(1)
N(11)	22(1)	31(1)	20(1)	1(1)	6(1)	1(1)
N(12)	20(1)	25(1)	20(1)	1(1)	8(1)	-2(1)
C(12)	17(1)	22(1)	22(1)	0(1)	5(1)	1(1)
C(13)	18(1)	24(1)	24(1)	0(1)	8(1)	0(1)
N(13)	21(1)	34(1)	18(1)	-1(1)	6(1)	-6(1)
C(14)	19(1)	26(1)	23(1)	1(1)	7(1)	-2(1)
C(15)	39(1)	70(1)	20(1)	-4(1)	10(1)	-23(1)
C(16)	22(1)	30(1)	28(1)	2(1)	12(1)	-2(1)
C(24)	26(1)	21(1)	24(1)	0(1)	10(1)	-4(1)
C(23)	33(1)	26(1)	28(1)	-2(1)	16(1)	-4(1)
C(22)	28(1)	27(1)	43(1)	-2(1)	18(1)	0(1)
O(22)	33(1)	35(1)	24(1)	3(1)	10(1)	4(1)
N(22)	24(1)	30(1)	25(1)	1(1)	10(1)	3(1)
C(21)	26(1)	27(1)	32(1)	0(1)	11(1)	-1(1)
N(21)	35(1)	33(1)	39(1)	-4(1)	22(1)	0(1)
O(21)	44(1)	53(1)	25(1)	-2(1)	17(1)	4(1)
C(20)	27(1)	20(1)	26(1)	-1(1)	13(1)	-4(1)
N(20)	25(1)	24(1)	27(1)	-1(1)	11(1)	-2(1)
F(20)	31(1)	51(1)	35(1)	3(1)	7(1)	9(1)

Table S8. Hydrogen coordinates (x 10^4) and isotropic displacement parameters (Å^{2}x 10 ^{3}) for THP:FAV.

	**x**	**y**	**z**	**U(eq)**
H(10N)	4987(14)	390(20)	-6060(12)	36(4)
H(151)	8192(16)	3980(30)	-2865(14)	55(6)
H(152)	8680(20)	1900(30)	-2770(16)	71(7)
H(153)	7476(18)	2290(30)	-2794(15)	65(7)
H(161)	9240(20)	2970(30)	-5963(16)	27(6)
H(162)	9017(17)	4850(30)	-5782(14)	16(5)
H(163)	8258(17)	3890(30)	-6738(16)	18(5)
H(164)	9465	3906	-5555	58(17)
H(165)	8385	4875	-6423	40(13)
H(166)	8747	2812	-6579	14(9)
H(10)	5197(13)	950(20)	-7599(12)	34(4)
H(23N)	5506(14)	4420(20)	989(11)	37(4)
H(22N)	6507(14)	3500(20)	1671(13)	35(4)
H(22)	10012(13)	420(20)	868(11)	33(4)
H(21O)	6678(19)	3080(30)	-1096(16)	67(7)

Table S9. Hydrogen bonds with H..A < r(A) + 2.000 Angstroms and <DHA > 110 deg for THP:FAV.

**D-H**	**d(D-H)**	**d(H..A)**	**<DHA**	**d(D..A)**	**A**
N10-H10N	0.891	1.877	169.77	2.758	O10 [-x+1, -y, -z-1]
C15-H153	0.924	2.27	110.4	2.733	O10
C15-H153	0.924	2.512	131.07	3.196	O21
C16-H164^b	1.083	2.613	121.32	3.308	O11 [-x+2, -y+1, -z-1]
C16-H166^b	1.078	2.45	156.35	3.464	F20 [x, y, z-1]
C10-H10	0.95	2.237	161.37	3.152	O11 [x-1/2, -y+1/2, z-1/2]
N22-H23N	0.898	2.161	167.78	3.044	O22 [-x+1, -y+1, -z]
N22-H22N	0.89	2.27	151.79	3.083	N11 [x, y, z+1]
C22-H22	0.966	2.569	146.28	3.414	N21 [-x+2, -y, -z]
O21-H21O	0.925	1.699	154.55	2.565	O22

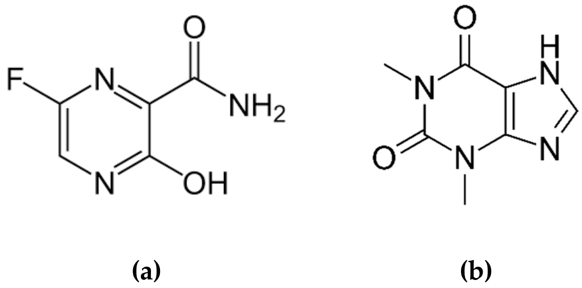

Figure S1. Chemical structures of the two APIs **(a)** Favipiravir and **(b)** Theophylline.

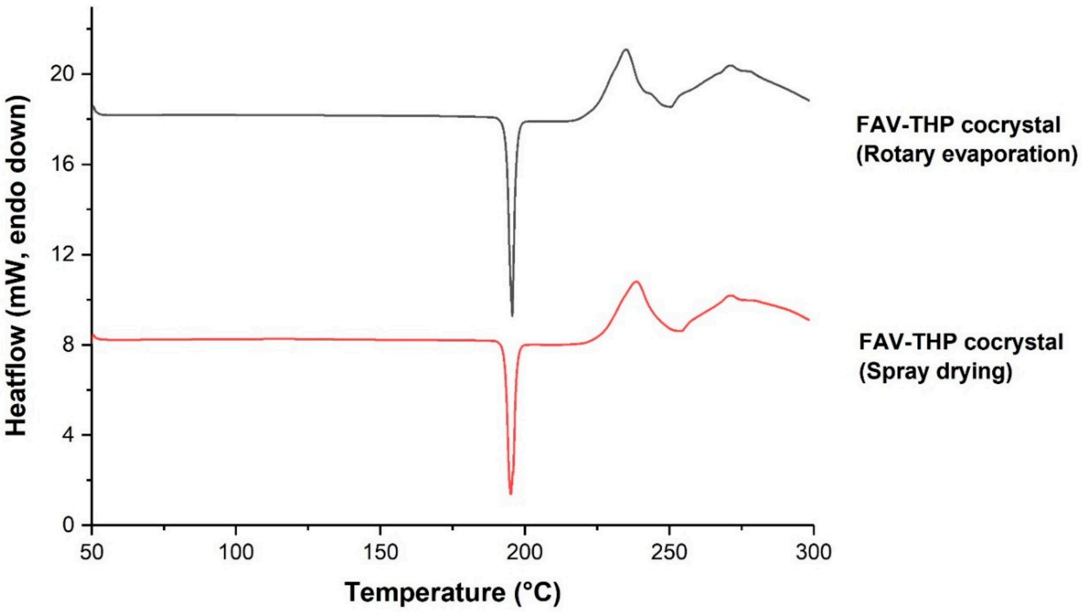

Figure S2. DSC thermograms of the FAV-THP cocrystal produced by rotary evaporation and spray drying after stability test.

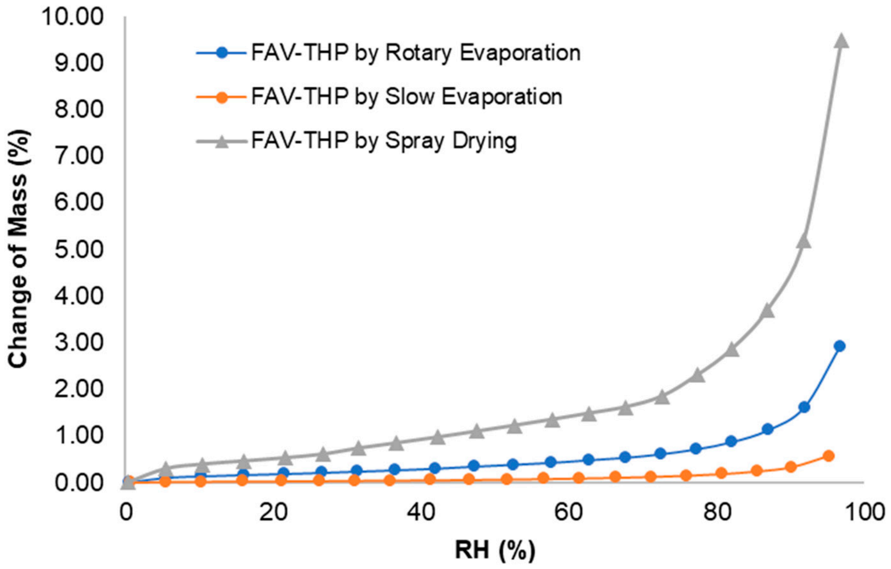

Figure S3. Water sorption isotherms at 25 °C of the FAV-THP cocrystals produced by different methods.

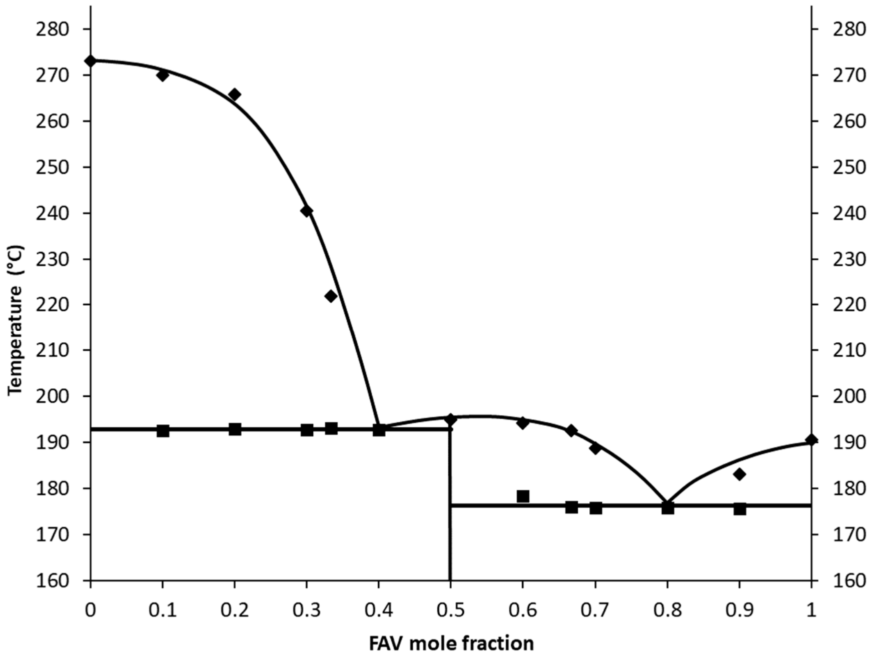

Figure S4. Temperature–composition phase diagram of the FAV-THP cocrystal system.

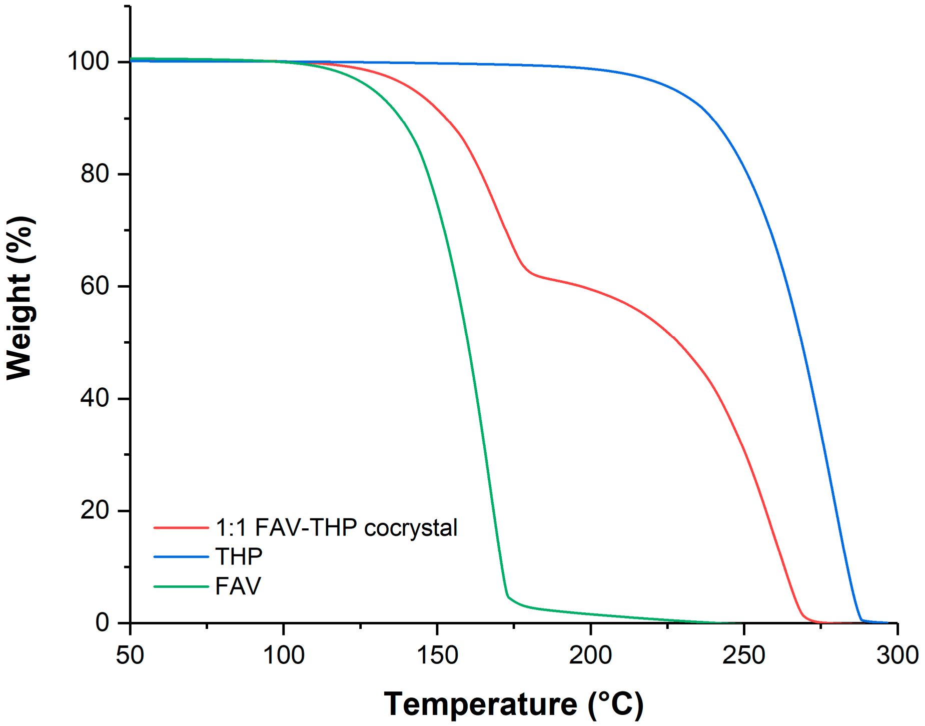

Figure S5. TGA profiles of the FAV-THP cocrystal system.

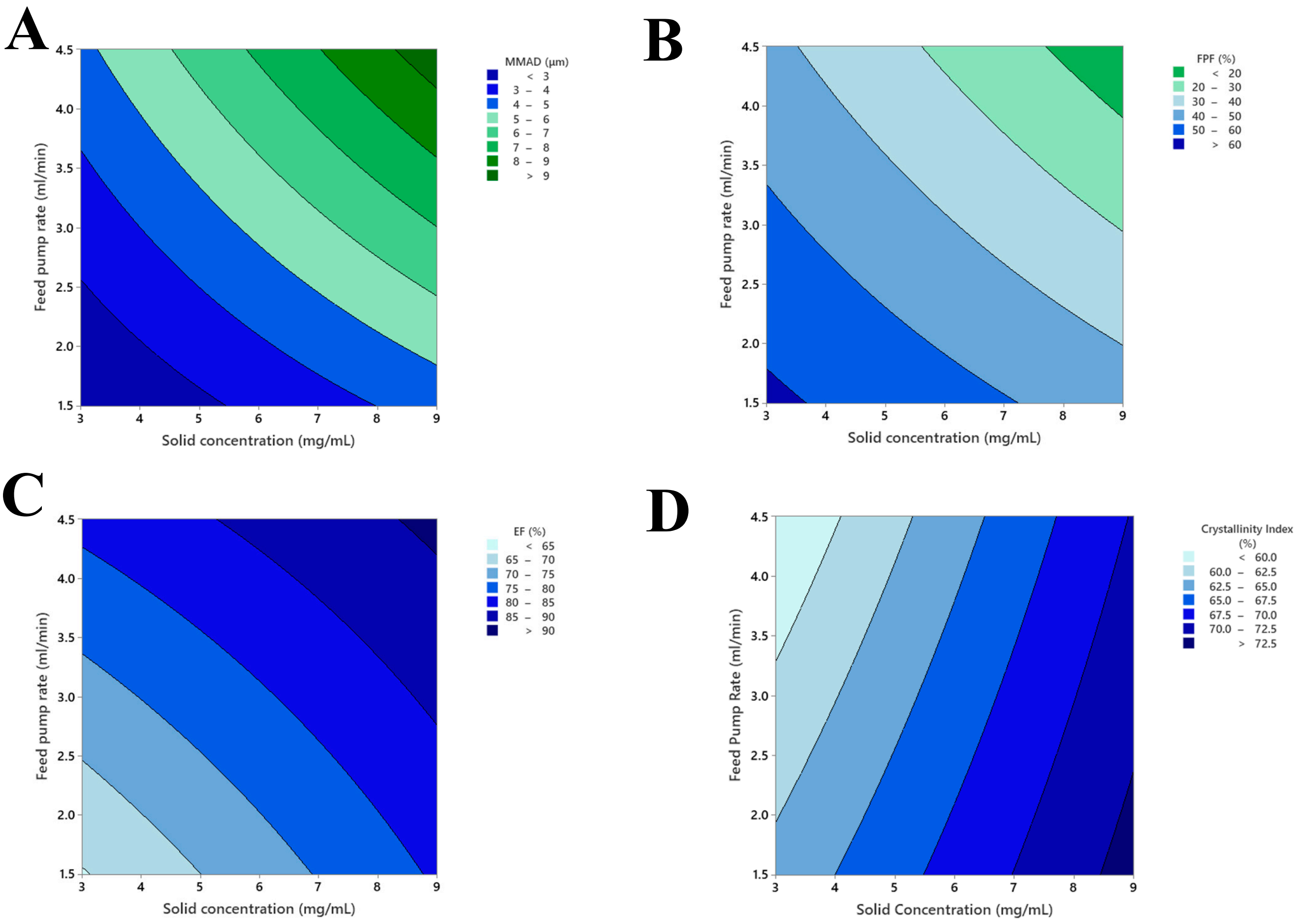

Figure S6. 2D contour plots for the (A) MMAD, (B) FPF, (C) EF, and (D) CI as projections of the total solute concentration (x-axis) and the feed pump rate (y-axis), holding a high level atomizing gas flow. The blue color represents areas within the design space where the defined limits, MMAD of <5 µm, FPF of ≥30%, EF of ≥60% and CI of ≥50%, are fulfilled.

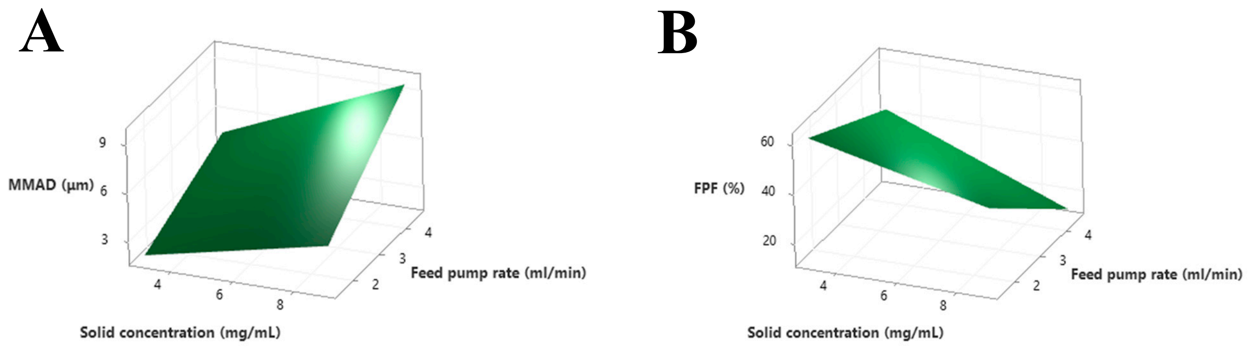

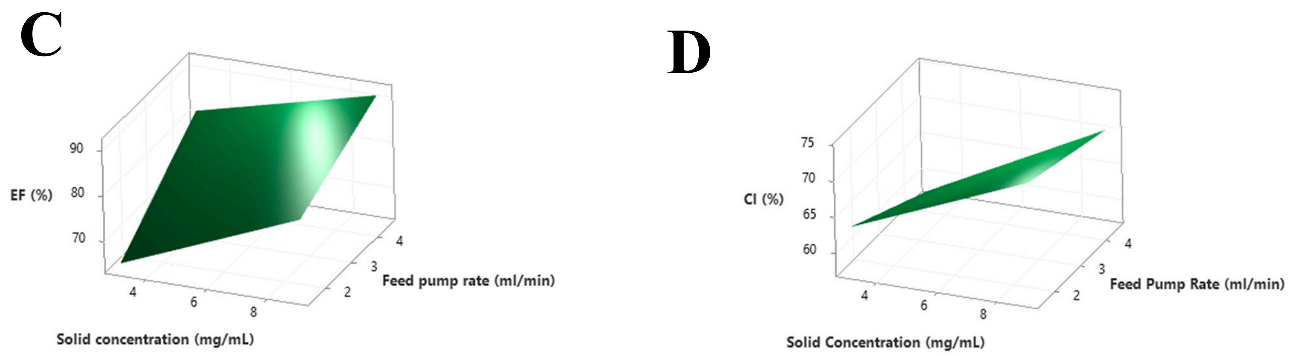

Figure S7. 3D surface plots for the (A) MMAD, (B) FPF, (C) EF, and (D) CI at high level atomizing gas flow.

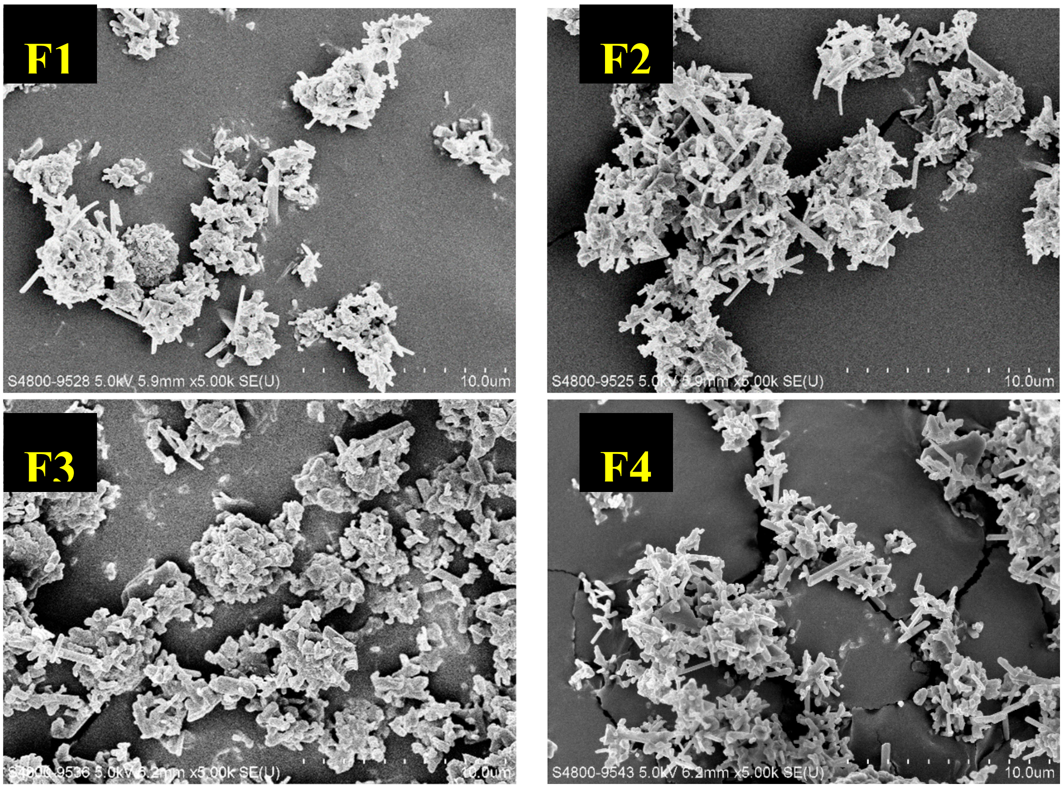

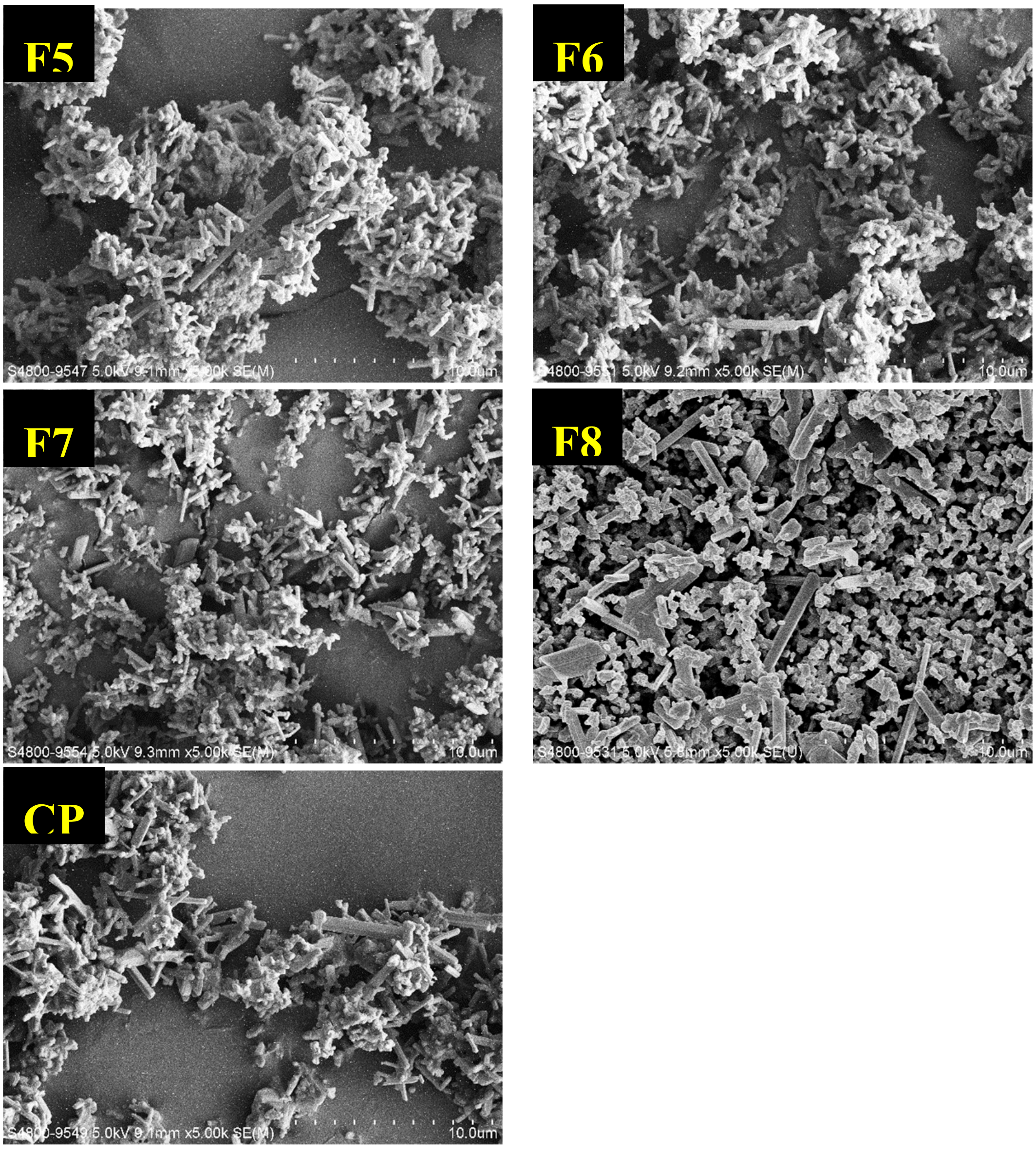

Figure S8. Scanning electron micrographs of different spray-dried cocrystal dry powder formulations at 5000× magnification.

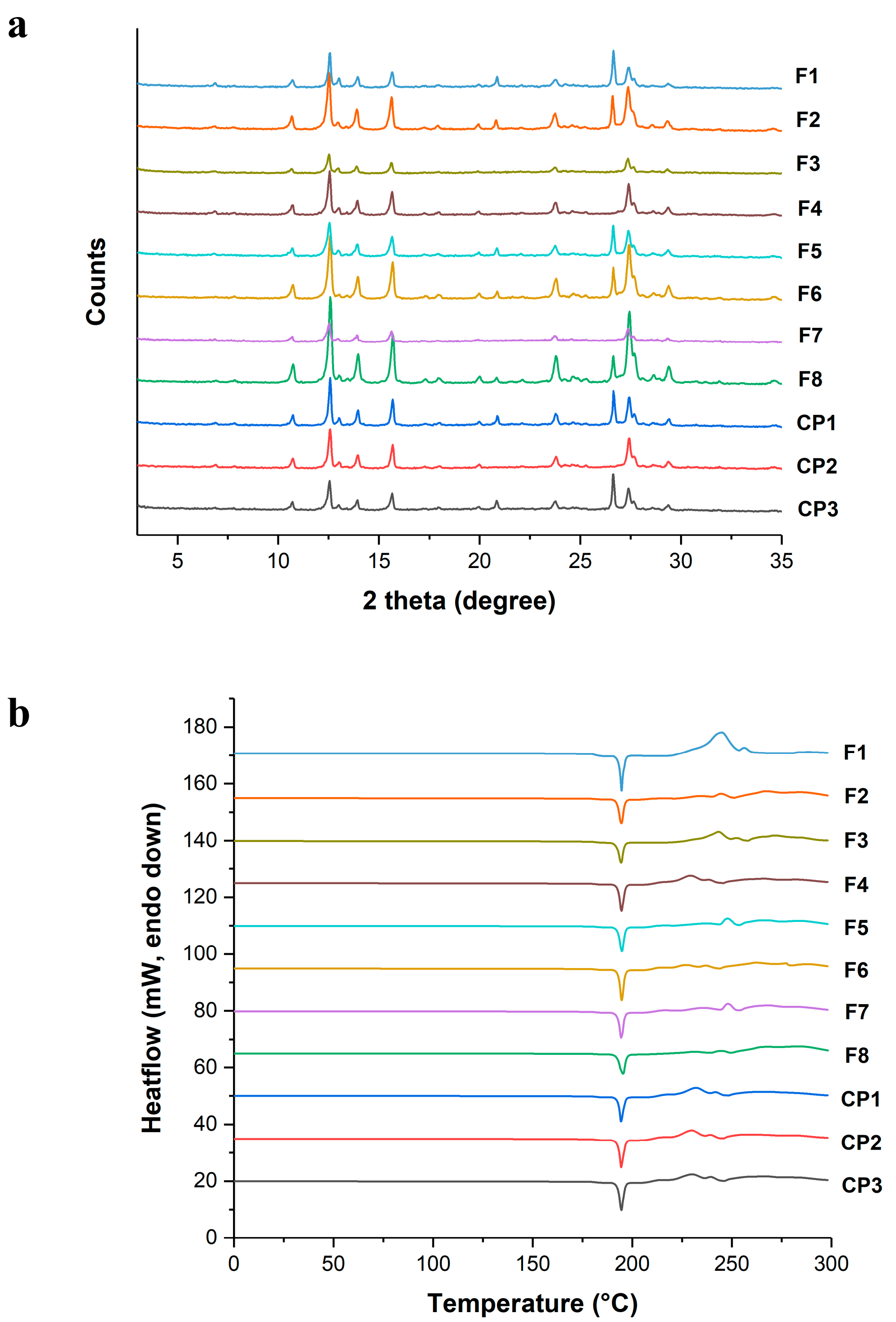

Figure S9. PXRD (**a**) and DSC (**b**) profiles of different spray-dried FAV-THP formulations.
